# Widely Tunable SNAP Microresonators Induced at the Optical Fiber Intersections

**DOI:** 10.1002/nap2.70366

**Published:** 2026-11-13

**Authors:** M. Sumetsky

**Affiliations:** ^1^ Aston Institute of Photonic Technologies Aston University Birmingham UK

## Abstract

A widely tunable free spectral range (FSR) is essential for many microresonator applications, but achieving it in compact high‐Q systems remains a significant challenge. Surface nanoscale axial photonics (SNAP) provides a route to such devices by localizing whispering‐gallery modes through nanoscale effective‐radius variations along an optical fiber. Recently, it has been experimentally demonstrated that side‐coupling between two optical fibers can induce high‐Q SNAP microresonators. In contrast to broadly explored monolithic microresonators, this configuration enables extensive mechanical tuning of the microresonator configuration and FSR through fiber bending, tilting, and twisting. Here, we develop the theory of SNAP microresonators induced at optical fiber intersections, which has remained largely unexplored. We consider weakly twisted fibers, whose geometry can be decomposed into tilting and bending. We show that extremely small fiber curvature critically affects the coupling‐induced cutoff frequency variation, the shape of the effective axial potential, and the spectrum of the induced resonator. We discuss the physical origin of this curvature and show that accounting for it resolves the discrepancy between theory and experiment. These findings establish side‐coupled optical fibers as a simple and reconfigurable platform for realizing microresonators with a widely tunable FSR.

## Graphical abstract

Optical fiber intersections create high‐Q microresonators with a widely tunable free spectral range. The theory reveals how inter‐fiber coupling localizes whispering‐gallery modes and how dramatically small fiber curvature reshapes the resonator spectrum. Accounting for this subtle geometric effect explains experimental observations and provides a framework for designing mechanically reconfigurable photonic microresonators.

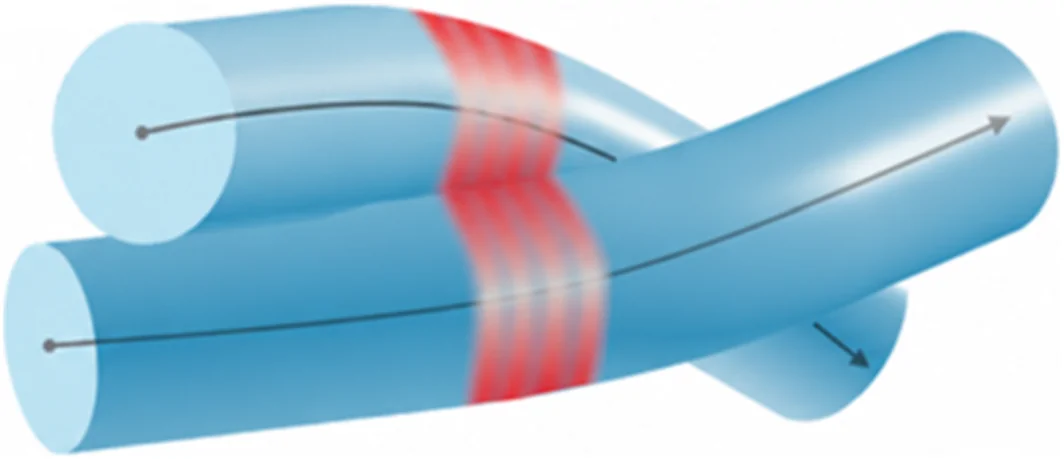

## 1 Introduction

Optical microresonators are fundamental components of modern photonic devices due to their compact size, high quality factors, and strong optical confinement. They have enabled numerous applications including optical filtering, sensing, nonlinear optics, frequency comb generation, optomechanics, and cavity quantum electrodynamics [1, 2, 3, 4, 5]. In many of these applications, including cavity electrodynamics [6, 7, 8], optical computing [9, 10], and optical frequency comb generation [11, 12], precise control of the resonator spectrum—particularly the free spectral range (FSR)—is of primary importance. However, in most monolithic whispering‐gallery‐mode (WGM) resonators such as microspheres, microtoroids, and microrings, the FSR is determined mainly by the resonator geometry and therefore remains essentially fixed after fabrication. Thermal, mechanical, and electro‐optic tuning techniques can shift resonance frequencies but typically modify them simultaneously, leaving their spacing nearly unchanged [6, 13, 14, 15, 16]. Achieving substantial and controllable FSR tunability in compact high‐Q resonators, therefore, remains a significant challenge.

Surface nanoscale axial photonics (SNAP) provides a unique platform for the realization of high‐Q microresonators based on nanoscale deformation of optical fibers [17]. In SNAP structures, WGMs, which circulate near the fiber surface and slowly propagate along the fiber axis, can be localized by nanoscale variation of the effective fiber radius. This variation is proportional to the variation of the WGM cutoff frequencies (CFs) $\omega_{m,q}^{\left(cut\right)}\left(z\right)$ along the fiber axis z, where m and q are the WGM azimuthal and radial quantum numbers. CFs act as *effective potentials* that confine WGMs along the fiber axis. Remarkably, variation in the effective radius on the nanometer scale can confine light along the fiber axis and form optical microresonators with high quality factors and unprecedented sub‐angstrom fabrication precision [17, 18, 19, 20, 21, 22, 23, 24, 25].

Several techniques have been developed to create SNAP microresonators through controlled modification of optical fibers. Early demonstrations employed localized annealing of the fiber surface using a CO_2_ laser, which relaxes frozen‐in stresses and produces nanoscale radius variations along the fiber axis [17, 18, 19, 20, 21]. Additional fabrication methods, including nanoscale modification of microcapillary fiber surface by heated water [22], femtosecond‐laser inscription [23], as well as flame and metal wire heating [24, 25] have also been used to introduce SNAP microresonators on optical fibers.

Recently, mechanical deformation of optical fibers was demonstrated as a particularly simple approach to creating tunable SNAP resonators. It was shown that strong bending of an optical fiber produces geometric deformation together with elasto‐optic refractive‐index variation, which generates an effective radius variation capable of localizing WGMs [26, 27]. This mechanism enables mechanically tunable microresonators whose spectral characteristics can be controlled by the fiber curvature. Controlled bending has been used to realize SNAP resonators with parabolic axial profiles and tunable picometer‐scale FSR [27]. A miniature tunable optical delay line based on this mechanism has been demonstrated [28].

Another important mechanism for inducing SNAP resonators is based on coupling between nearby optical fibers (Figure 1). It has been shown that side‐coupled fibers can produce localized variations of the CF that form high‐Q microresonators in the coupling region [29]. In this configuration, the resonator length and spectral characteristics can be tuned by adjusting the fiber curvature, enabling wide tuning of the microresonator FSR (Figure 1a). Such mechanically reconfigurable resonators are promising for applications including tunable optical delay lines, frequency comb generators, and tunable miniature photonic circuits.

**FIGURE 1:**
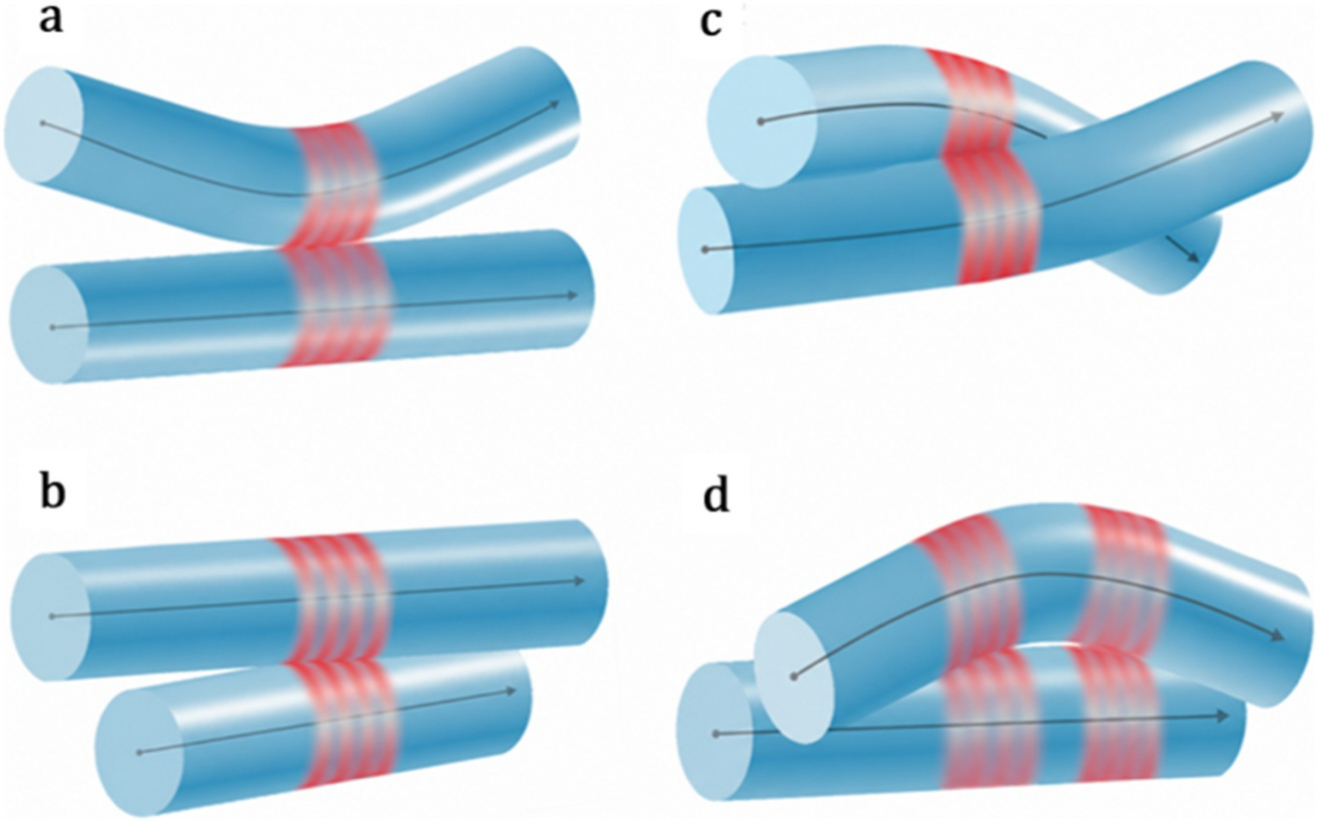
(a) Bent, (b) tilted, and (c) twisted fiber configurations. (d) Bent fiber configuration with two contact points.

More recently, an even simpler configuration has been demonstrated experimentally: microresonators formed at the intersection of two straight optical fibers [30] (Figure 1b). In this geometry, coupling between the fibers produces a localized variation of the CFs, which in turn localize WGMs slowly propagating along the fiber axis. Remarkably, extremely small relative tilting of the fiber segments by milliradian angles, which can be introduced by micrometer‐scale translations of their ends, is sufficient to reshape this effective potential over millimeter‐scale distances and produce substantial tuning of the resonator spectrum, including order‐of‐magnitude variation of the free spectral range [30]. This configuration is particularly attractive because it minimizes mechanical stress and enables compact implementations potentially compatible with MEMS technologies. Additionally, in Ref. [31] FSR tunable microresonators were demonstrated by parallel translation of optical fibers. The latter approach achieves better tuning precision at the expense of a larger translation range. Finally, preliminary experimental observations of microresonators induced in strongly twisted fiber configurations were reported in Ref. [32].

Despite the experimental demonstration of the FSR tunable microresonator configurations noted above [29, 30, 31, 32], the physical mechanisms governing light localization in intersecting optical fibers, and in particular, the role of the geometric parameters of the considered fiber configurations were not well understood. Consequently, the correspondence of the experimental data with the simplified theory developed to date remained limited (see, e.g., Refs. [29, 30]).

In this work, we develop the theory of optical microresonators induced at the intersections of *weakly twisted* optical fibers, whose geometry can be decomposed into *tilting and bending*. The axial localization of WGMs in this configuration can be described by coupled wave equations governing the slow axial propagation of WGMs determined by the CF variation along the fiber axis. We show that the extremely small though unavoidable curvature of fibers plays a crucial role in determining the shape of induced microresonators and their spectra. Taking this curvature into account allows us to achieve good agreement between the developed theory and the experimental results.

## 2 Results

### 2.1 Geometrical Description of the Twisted Fiber Configuration

We consider two twisted optical fibers with the same radius $r_{0}$ illustrated in Figure 2 where we introduce Cartesian coordinates (x,y,z). We choose the axis z to be approximately aligned with the axes of fibers 1 and 2, and consequently, axes x and y to be approximately in the transverse plane. The equations defining the axes of these fibers have the form $\left(x_{j}\left(z\right),y_{j}\left(z\right),z\right)$, j=1,2. We are interested only in the small vicinity of the region where the fibers contact each other. In the vicinity of this region, the fiber axis profiles can be approximated by *quadratic dependencies on* z. In this approximation, both fiber axes have *zero torsion*, which is proportional to the third derivative, and therefore experience *planar bending* [33]. Under these assumptions, the equations describing the slightly curved fiber axes in the region of their proximity can be written as follows:

(1) $$\begin{array}{l} x_{1}\left(z\right)=-r_{0}-\frac{\Delta h_{x}}{2}+\frac{z^{2}}{2R_{1}}\\ y_{1}\left(z\right)=-\frac{\alpha z}{2}\\ x_{2}\left(z\right)=r_{0}+\frac{\Delta h_{x}}{2}+\frac{z^{2}}{2R_{2}}\\ y_{2}\left(z\right)=\frac{\alpha z}{2} \end{array}$$

**FIGURE 2:**
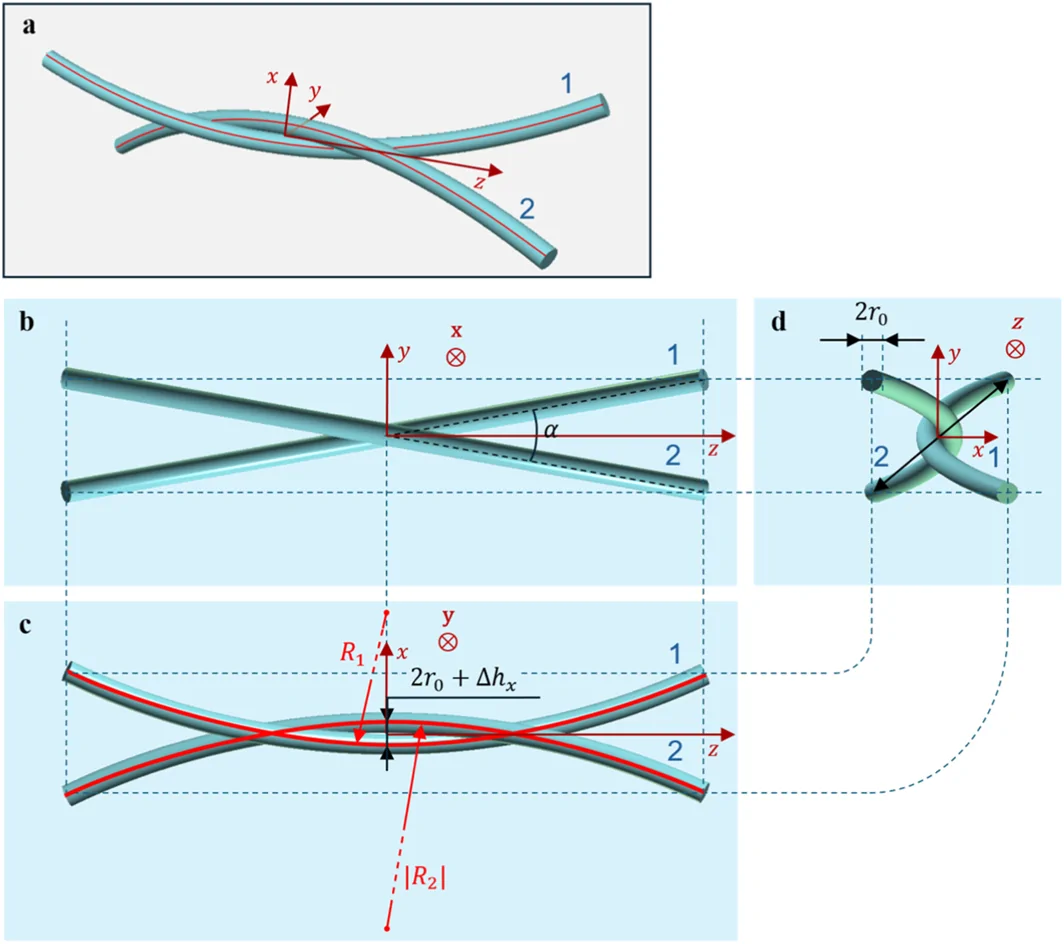
Twisted fiber configuration. (a) 3D figure. (b, c, and d) Projections of figure (a) onto (y,z), (x,z), and (x,y) planes, respectively.

where ${\Delta h}_{x}>0$, since the fibers cannot penetrate one another. Figure 2b,c,d show the mutually twisted fiber views along the plane normal to the axis x, y, and z, respectively. To keep the coupling length between the fibers much longer than their radius $r_{0}$, we assume that the tilt angle is small, α≪1, (Figure 2b) and the bending radii of fibers are much greater than the fiber radius, $|R_{j}|\gg r_{0}$ (Figure 2c). More generally, we assume that, in the region of our interest, where the inter‐fiber coupling may be significant, the deviation of the fiber axes in the coordinate plane (x,y) is relatively small compared to the fiber radius $r_{0}$, that is, $|x_{j}\left(z\right)-x_{j}\left(0\right)\left|,\right|y_{j}\left(z\right){-y}_{j}\left(0\right)|\ll r_{0}$.

The distance between the fiber surfaces as a function of the coordinate z can be expressed through the distance between fiber axes d(z) as follows:

(2) $$\Delta d\left(z\right)=d\left(z\right)-2r_{0}=\sqrt{{\left(x_{1}\left(z\right)-x_{2}\left(z\right)\right)}^{2}+{\left(y_{1}\left(z\right)-y_{2}\left(z\right)\right)}^{2}}-2r_{0}.$$

We are only interested here in the fiber inter‐surface distance Δd(z) which is small compared to the fiber radius $r_{0}$. Furthermore, to enable inter‐fiber mode coupling, this distance should be of the order of a micron or less. Under these assumptions and using Equation (1), we transform Equation (2) to the following:

(3) $$\Delta d\left(z\right)=\Delta h_{x}+\frac{1}{2}\left(\frac{1}{R_{eff}}+\frac{\alpha^{2}}{2r_{0}}\right)z^{2},R_{eff}={\left(\frac{1}{R_{2}}-\frac{1}{R_{1}}\right)}^{-1}.$$

where the effective fiber curvature radius $R_{eff}$ is introduced.

The twisted fiber configuration shown in Figure 2 reduces to the coplanar bent geometry illustrated in Figure 1a [29] when the tilt angle vanishes (α=0), and to the tilted straight‐fiber geometry illustrated in Figure 1b [30] when the fiber curvatures vanish ($R_{j}=\infty$). As illustrated in Figure 1, in the quadratic approximation considered the fibers may have one, two, or no contact points. In degenerate cases, they may contact over a curve that jointly belongs to both fibers. In this paper, we restrict our analysis to the case of a single minimum ${\Delta h}_{x}$ of the inter‐fiber separation illustrated in Figure 2, when the case shown in Figure 1d is excluded.

### 2.2 Microresonators Induced at Fiber Intersections: Cutoff Frequency Resonance

The wave equation, which describes WGMs E(r) *slowly propagating* along the configurations of fibers illustrated in Figure 1 and described by Equations (1)‐(3) can be written down as follows:

(4) $$\Delta E+\frac{\omega^{2}}{c^{2}}n^{2}\left(r\right)E=0$$

where n(r) is the refractive index of the considered system, c is the speed of light, and ω is the angular frequency of light. The WGMs in each of the *uncoupled* weakly bent fibers with the number j=1,2 can be presented as $E_{j}^{\left(0\right)}\left(r\right)=\exp\left(i\beta_{j}\left(\omega \right)s_{j}\right)\exp\left(im_{j}\theta_{j}\right)Q_{j}\left(\rho_{j}\right)$, where $\left(\rho_{j},\theta_{j},s_{j}\right)$ is the local cylindrical coordinate system along the axis $s_{j}$ of fiber j. For the WGM frequencies close to the CFs $\omega_{c,j}$, the mode propagation constant as a function of light frequency ω can be expressed in each of the fibers as follows: (see, e.g., [17])

(5) $$\beta_{j}\left(\omega \right)=\frac{n_{0}}{c}\sqrt{2\omega_{0}\left(\omega -\omega_{c,j}\right)},$$

where $n_{0}$ is the fiber refractive index and $\omega_{0}≅\omega_{cj}$. For a small coupling between fibers, the WGMs in each of them can be presented as $E_{j}\left(r\right)=\Psi_{j}\left(z\right)\exp\left(im_{j}\theta_{j}\right)Q_{j}\left(\rho_{j}\right)$, where the dependence along the axis z in fiber j is described by functions $\Psi_{j}\left(z\right)$ satisfying the coupled wave equations (see Supporting Information S1: Sections S1 and S2):

(6) $$\begin{array}{l} \frac{d^{2}\Psi_{1}}{dz^{2}}+\beta_{1}^{2}\left(\omega \right)\Psi_{1}=-\frac{\omega_{0}^{2}}{c^{2}}I_{12}\left(z\right)\Psi_{2}\\ \frac{d^{2}\Psi_{2}}{dz^{2}}+\beta_{2}^{2}\left(\omega \right)\Psi_{2}=-\frac{\omega_{0}^{2}}{c^{2}}I_{21}\left(z\right)\Psi_{1} \end{array}$$

where $I_{ij}$ are the coupling coefficients. For the twisted fiber configuration considered, we find (see Supporting Information S1: Sections S1 and S2):

(7) $$\begin{array}{l} I_{12}\left(z\right)=I_{21}^{*}\left(z\right)=I_{0}\;\exp\left[i\beta_{0}z-\sigma d\left(z\right)\right],\\ I_{0}≃\frac{2\pi^{-1/2}{\left(-1\right)}^{q}n_{0}^{2}}{{\left(n_{0}^{2}-1\right)}^{3/4}}{\left(\frac{c}{\omega_{0}r_{0}}\right)}^{3/2},\beta_{0}=\frac{2\pi \alpha n_{0}}{\lambda_{0}},\sigma =\frac{\omega_{0}{\left(n_{0}^{2}-1\right)}^{1/2}}{c}, \end{array}$$

where q is an integer equal to the sum of the radial quantum numbers of fiber modes and the effective fiber curvature radius $R_{eff}$ and separation between fibers Δd(z) are defined in Equation (3). The flux conservation law, which simply follows from Equations (6) and (7), shows that, in the approximation considered, the coupling‐induced losses are absent.

From Equations (6) and (7), the spatial variation of the coupling coefficients $I_{ij}$ is determined by the characteristic distance $\beta_{0}^{-1}$ inverse proportional to the tilt angle α and characteristic distance $\sigma^{-1}$. As expected, the coupling coefficients $I_{ij}$ exponentially decrease with the separation between fibers Δd(z). We find from Equation (3) for Δd(z) that for the positive $R_{eff}$, the fiber curvatures $1/R_{j}$ and tilt angle α are jointly responsible for the localization of coupling and for reducing the induced microresonator's axial size. However, for negative $R_{eff}$, the curvature compensates for the tilt at $R_{eff}=-2r_{0}\alpha^{-2}$. The latter relation between the effective radius and tilt angle is plotted for $r_{0}=20\;\mu m$, $r_{0}=62.5\;\mu m$, and $r_{0}=100\;\mu m$ in Figure 3. For $R_{eff}<0$ and $\left|R_{eff}\right|<2r_{0}\alpha^{-2}$, the single‐point inter‐fiber contact configuration can evolve into two inter‐fiber point contacts or a contact along a continuous line segment joining the fiber surfaces whose length can be enhanced by the van der Waals attraction [30, 34, 35].

**FIGURE 3:**
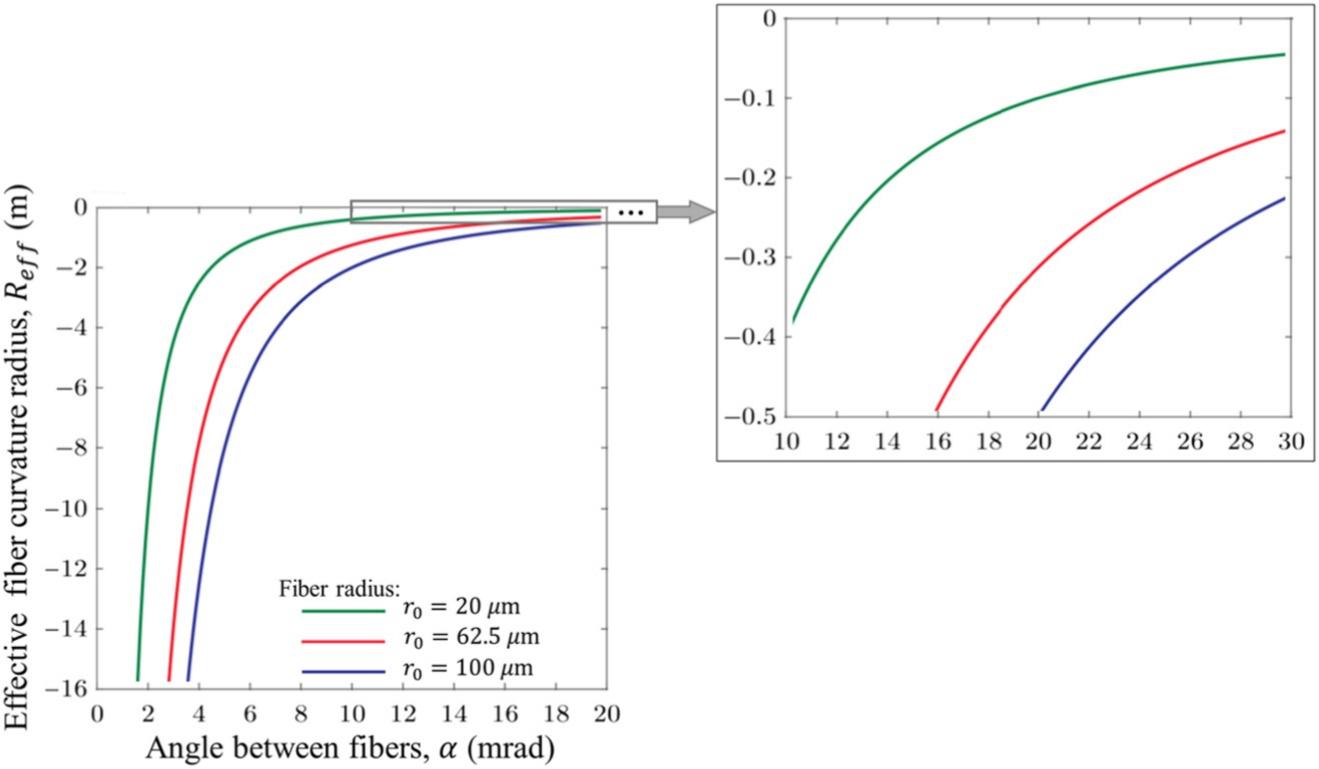
The relation between the tilt angle α and the negative effective curvature radius $R_{eff}=-2r_{0}\alpha^{-2}$ compensating for this tilt for $r_{0}=20$ μm (green curve), $r_{0}=62.5$ μm (red curve), and $r_{0}=100$ μm (blue curve).

It is shown in Supporting Information S1: Section S3 that CFs $\omega_{c}^{\pm }\left(z\right)$ of the coupled fiber system considered can be found in the WKB approximation as follows:

(8) $$\omega_{c}^{\pm }\left(z\right)=\frac{1}{2}\left(\omega_{c,1}+\omega_{c,2}\pm \sqrt{{\left(\omega_{c,1}-\omega_{c,2}\right)}^{2}+\frac{\omega_{0}^{2}}{n_{0}^{4}}I_{12}\left(z\right)I_{21}\left(z\right)}\right)$$

The characteristic dependencies of $\omega_{c}^{\pm }\left(z\right)$ near the coupling region are illustrated in Figure 4, which exhibits the well‐known effect of coupling‐induced level repulsion [36]. As a result of repulsion, the larger CF, $\omega_{c}^{+}\left(z\right)$, is bent up and forms a potential barrier, while the smaller one, $\omega_{c}^{-}\left(z\right)$, is bent down and forms a quantum well, that is, a microresonator induced by fiber coupling. As shown in Supporting Information S1: Section S3, the propagation constants of the system (now depending on the axial coordinate z) can be expressed through the CFs $\omega_{c}^{\pm }\left(z\right)$ similar to Equation (5) as follows:

(9) $$\beta_{\pm }^{2}\left(z,\omega \right)=2\omega_{0}\frac{n_{0}^{2}}{c^{2}}\left(\omega -\omega_{c}^{\mp }\left(z\right)\right).$$

**FIGURE 4:**
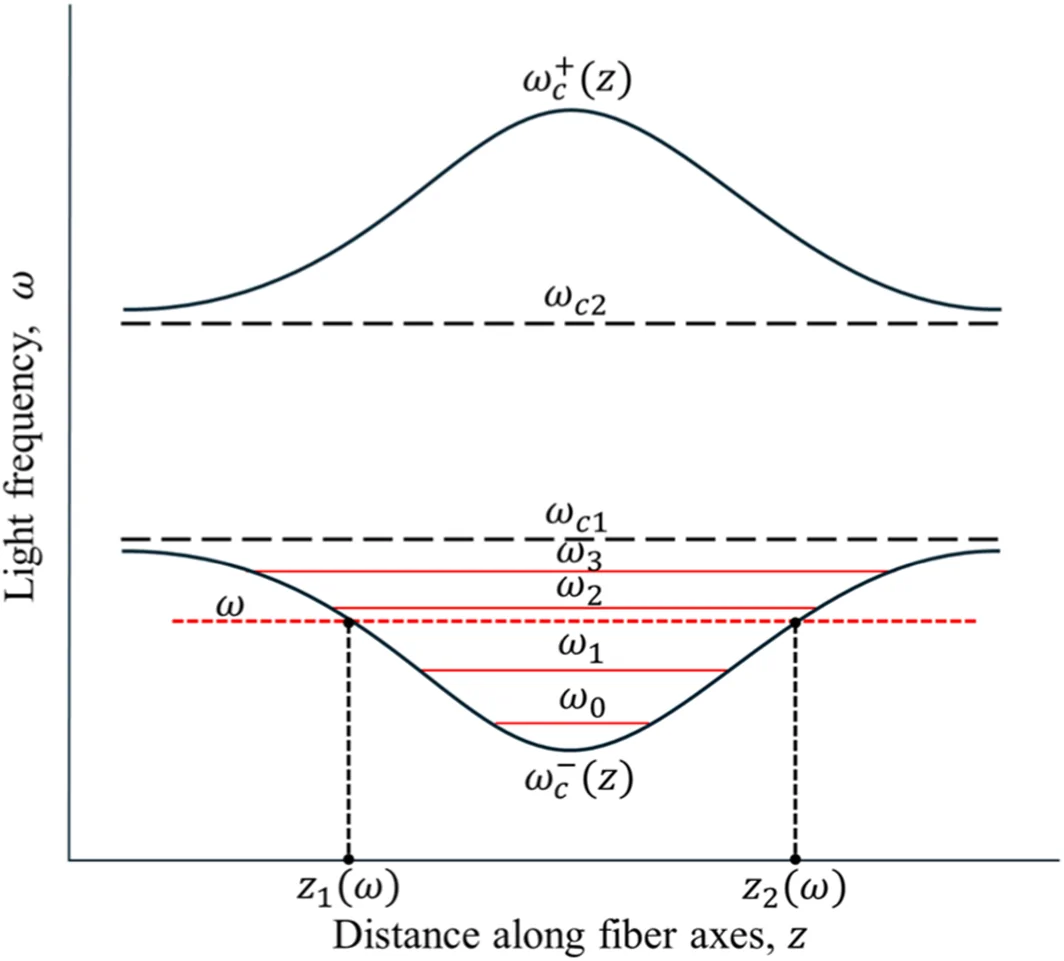
Behavior of CFs $\omega_{c}^{\pm }\left(z\right)$ near the coupling region.

In the WKB approximation, for a known CF quantum well profile, $\omega_{c}^{-}\left(z\right)$, the resonator eigenfrequencies can be found from the Bohr–Sommerfeld quantization rule [36],

(10) $$\int_{z_{1}\left(\omega_{k}\right)}^{z_{2}\left(\omega_{k}\right)}\beta_{+}\left(z,\omega_{k}\right)dz=\pi \left(k+\frac{1}{2}\right),k\gg 1,\text{integer}.$$

Here, $z_{j}\left(\omega \right)$ are the WGM turning points (zeros of $\beta_{+}\left(z,\omega \right)$) shown in Figure 4.

The asymptotic expression for the coupling coefficients $I_{ij}$ in Equation (7) loses accuracy at small fiber separations, where the coefficients are largest and have the greatest effect on the CFs. Therefore, in Supporting Information S1: Section S4 we calculate these coefficients numerically using the multipole method. In the adiabatic approximation, which is valid for small tilt angles α and large bending radii $R_{eff}$ considered here, we find the local CFs as eigenfrequencies of the two‐dimensional coupled‐fiber cross‐section, calculated as functions of the fiber separation $\Delta d\left(z\right)=\Delta h_{x}+\frac{1}{2}\left(R_{eff}^{-1}+\alpha^{2}{/2r}_{0}\right)z^{2}$ (see Equation (3)). The dependence of $I_{12}$ on α, $R_{eff}$, and z is incorporated in $I_{12}$ through its dependence on Δd(z). At the exact matching of CFs of uncoupled fibers, $\omega_{c,1}=\omega_{c,2}$, the CF quantum well profile is found from Equation (8) as $\omega_{c}^{-}\left(z\right)=\omega_{c,1}-\frac{1}{2}\omega_{0}n_{0}^{-2}|I_{12}(\Delta d\left(z\right)|$. Similarly, for the coupling much smaller than the CF separation, $I_{0}\ll 2{n_{0}}^{2}\left|\omega_{c,2}-\omega_{c,1}\right|/\omega_{0}$, we arrive at the CF quantum well whose FWHM is $\sqrt{2}$ times smaller than that for the exact CF matching: $\omega_{c}^{-}\left(z\right)=\omega_{c,1}-\frac{1}{4}\omega_{0}^{2}n_{0}^{-4}{\left|\omega_{c,2}-\omega_{c,1}\right|}^{-1}{\left|I_{12}(\Delta d\left(z\right)\right|}^{2}$. For small fiber separation, when $\Delta d\left(z\right)≲c\omega_{0}^{-1}{\left(n_{0}^{2}-1\right)}^{-\frac{1}{2}}≅0.2$ μm for $\omega_{0}=2\pi \cdot 193$ THz and $n_{0}=1.44$, these approximations lose accuracy and have to be corrected by numerical calculations (see Supporting Information S1: Figures S6 and S7).

Comparison of the numerical calculation and analytical approximations of the CF splitting based on Equation (7) on Supporting Information S1: Figure S6 shows that their relative difference is less than 30% for Δd(z)<1 μm achieving zero at Δd(z)≅0.29 μm. Therefore, it is reasonable to use the analytical results of this section to find the estimates of the axial FWHM ${\Delta z}_{FWHM}$ of the induced resonator and also of the FSR ${\Delta \omega }_{FSR}$ near its bottom where the CF quantum well is close to parabolic. At the exact resonance, $\omega_{c,2}=\omega_{c,1}$, we find from Equations (7) and (8):

(11) $$\Delta z_{\text{FWHM}}=4{\left[\frac{\ln\left(2\right)cr_{0}}{{\left({n_{0}}^{2}-1\right)}^{1/2}\omega_{0}\left(\frac{2r_{0}}{R_{\text{eff}}}+\alpha^{2}\right)}\right]}^{1/2},\Delta \omega_{\text{FSR}}=\frac{c^{5/4}{\left(\frac{2r_{0}}{R_{\text{eff}}}+\alpha^{2}\right)}^{1/2}\exp\left[-\frac{\omega_{0}}{2c}{\left({n_{0}}^{2}-1\right)}^{1/2}\Delta h_{x}\right]}{2^{1/2}\pi^{1/4}n_{0}{\left({n_{0}}^{2}-1\right)}^{1/8}{\omega_{0}}^{1/4}{r_{0}}^{5/4}}.$$

Based on Equation (11), Figure 5 illustrates how fiber tilting and bending jointly control the axial width and FSR of the induced microresonator. In this figure we assume ${\Delta h}_{x}=0$ and exact CF resonance, when the depth of the CF quantum well calculated from Equations (7) and (8) is 25. 5 GHz compared to 19.7 GHz numerically calculated in SI. Increasing α narrows the resonator (Figure 5a) and increases its FSR (Figure 5b). At small tilt angles, fiber curvature determines the resonator width and a nonzero FSR. As $\alpha^{2}$ becomes much larger than $2r_{0}/R_{\text{eff}}$, tilting dominates and the curves for different curvature radii approach one another. In this regime, the FWHM decreases approximately as 1/α, whereas the FSR increases approximately linearly with α. Consequently, weakly curved fibers allow a broader relative tuning range, while stronger bending reduces the sensitivity to small variations of the tilt angle. Increasing ${\Delta h}_{x}$ to 0.2 μm, when, as noted above, the considered approximation becomes much more accurate, we find that the dependence of FWHM ${\Delta z}_{FWHM}$ does not change, whereas the FSR ${\Delta \omega }_{FSR}$ becomes 1.5 times smaller.

**FIGURE 5:**
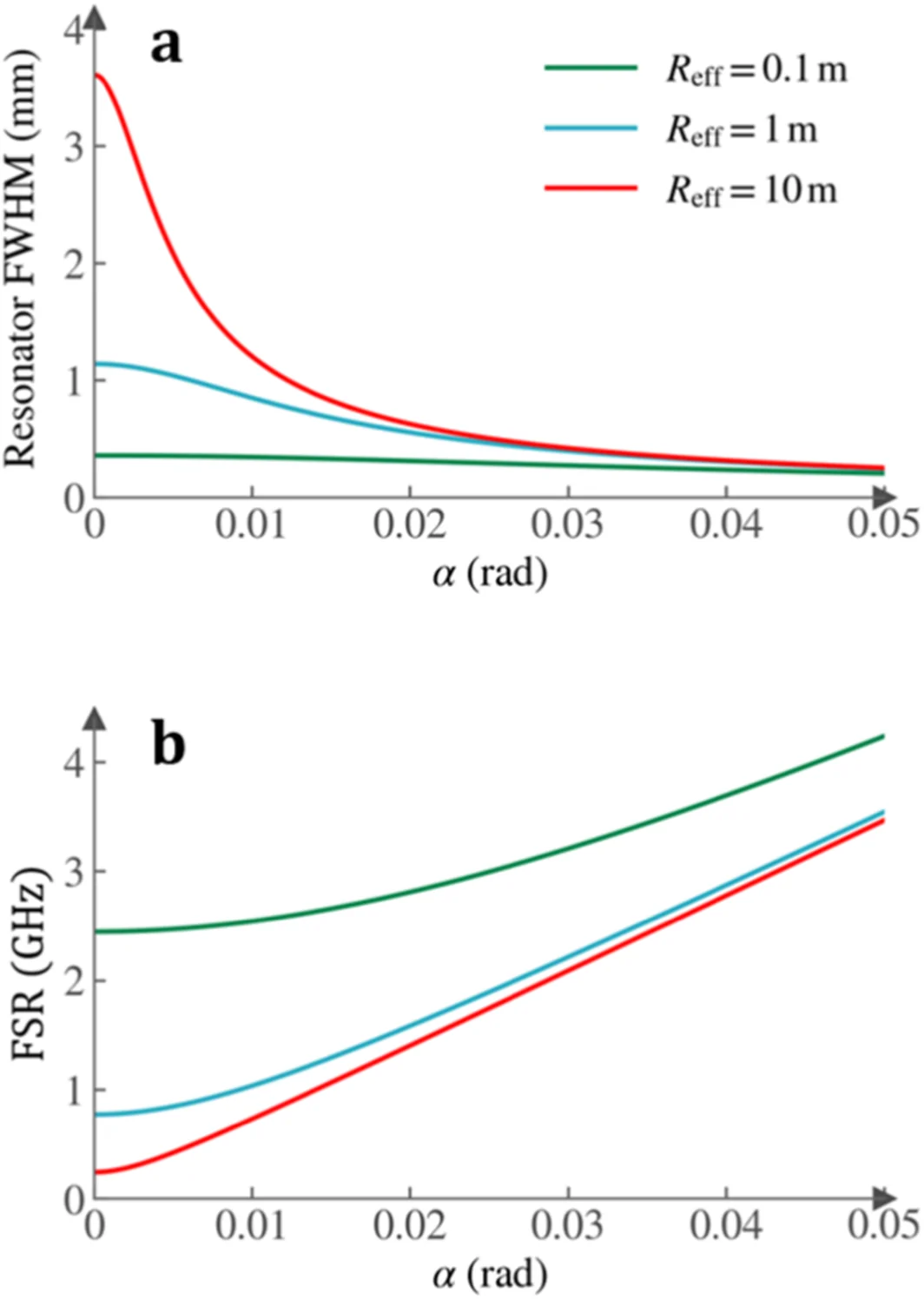
(a) Microresonator FWHM ${\Delta z}_{\text{FWHM}}$ and (b) FSR ${\Delta \omega }_{\text{FSR}}/\left(2\pi \right)$ as functions of the tilt angle α for effective curvature radii $R_{\text{eff}}=0.1$ m, 1 m, and 10 m. The curves are calculated from Equation (11) with $r_{0}=62.5$ μm, $n_{0}=1.45$, and $\omega_{0}/\left(2\pi \right)\approx 193.4$ THz.

Using Equation (11), we can estimate the achievable precision of experimental FSR tuning. For the parameters of Figure 5b, a rotation stage with an angular repeatability of 0.1 mrad limits the tilt‐induced FSR error to less than 10 MHz, provided that fiber curvature and contact conditions remain unchanged. Equivalent angular control can be obtained by lateral translation with 1 μm repeatability over a 10 mm lever arm. These requirements are readily achievable with precision stages. The FSR can also be tuned by adjusting the minimum inter‐fiber gap $\Delta h_{x}$. For the parameters of Figure 5b, increasing this gap by 0.1 μm reduces the predicted FSR by approximately 20%. With stable fiber mounting, the 10 nm positioning repeatability available with closed‐loop piezoelectric stages would correspond to approximately 2% FSR repeatability, or about 20 MHz for a 1 GHz FSR, provided that the fiber curvature and tilt remain unchanged. The silica thermal response shifts individual resonance frequencies by order 1 GHz/K near 1550 nm. Therefore, limiting this drift to 0.1 GHz requires temperature stability of approximately 0.1 K. A uniform temperature change shifts neighboring resonances together, with a much smaller effect on the FSR. Finally, fiber segments whose CFs match to within 0.1 GHz can be selected from the same parent fiber [37].

### 2.3 Microresonators Induced at Fiber Intersections: Cutoff Frequency Perturbation

In the previous Section, we considered resonant coupling between fibers with two closely spaced CFs, separated from all other CFs. Then, the CF shift due to coupling can be comparable to the separation between them. Now, we assume the case where the CFs of fibers are perturbed by their side‐coupling so that this shift is much smaller than the CF separation. Then, the variation of CFs is determined by the coupling of multiple transverse eigenstates of fibers. For weak inter‐fiber coupling, the WGMs in the fiber j can be presented as a linear combination $\sum_{p_{j}=1}^{P_{j}}Q_{j,p}\left(\rho_{j}\right)\exp\left(im_{j,p_{j}}\theta_{j}\right)\Psi_{j,p_{j}}\left(z\right)$ where $\Psi_{j,p_{j}}\left(z\right)$ are determined by the coupled wave equations of $P_{j}$ fiber modes, which are the direct generalization of Equation (6). For the uncoupled fiber j, we have $\Psi_{j,p_{j}}\left(z\right)∼\exp\left(i\beta_{j,p_{j}}\left(\omega \right)z\right)$, where $\beta_{j,p_{j}}\left(\omega \right)=\left(n_{0}/c\right){\left(2\omega \left(\omega -\omega_{c,j,p_{j}}\right)\right)}^{1/2}$ and $\omega_{cj,p_{j}}$ is a CF. (see Equation (5)). In the adiabatic approximation for coupled fibers, we set $\Psi_{j,p_{j}}\left(z\right)∼A_{j,p_{j}}\sum_{\;\pm }\exp\left(\pm \;i\int^{z}\;\beta \left(z^{\prime },\omega \right)dz^{\prime }\right)$, where $\beta \left(z,\omega \right)=\left(n_{0}/c\right){\left(2\omega \left(\omega -\omega_{c}\left(z\right)\right)\right)}^{1/2}$ and $\omega_{c}\left(z\right)$ is the CF of the coupled fibers. Then, the coupled wave equations are transferred to the system of linear algebraic equations

(12) $$\frac{2n_{0}^{2}}{\omega }\left(\omega_{c}\left(z\right)-\omega_{cj,p_{j}}\right)A_{j,p_{j}}+\sum_{p_{3-j}}I_{j,p_{j},p_{3-j}}\left(z\right)A_{3-j,p_{3-j}}=0,j=1,2,p_{j}=1,2,...,P_{j},$$

where $I_{j,p,q}$ are the coupling coefficients. For a small inter‐fiber coupling, when $\omega n_{0}^{-2}\left|I_{{j,p}_{j}{,p}_{3-j}}\right|$ is much smaller than the separation between the CFs of uncoupled fibers, $\left|\omega_{c,{j,p}_{j}}-\omega_{c{,3-j,p}_{3-j}}\right|$, the field amplitudes $A_{j,p_{j}}$ can be found by the perturbation theory. In particular, $\omega_{c}\left(z\right)$ is found by setting the determinant of this equation equal to zero, leading to the algebraic equation of the $P_{1}+P_{2}$‐order for $\omega_{c}\left(z\right)$. Assuming that one of the solutions, $\omega_{c}^{\left(p_{j}\right)}\left(z\right)$, is close to the unperturbed eigenfrequency $\omega_{c,{j,p}_{j}}$, we find the following:

(13) $$\omega_{c}^{\left(p_{j}\right)}\left(z\right)=\omega_{c,j,p_{j}}+\frac{\omega_{0}^{2}}{4n_{0}^{4}}\sum_{p_{3-j}=1}^{P_{3-j}}\frac{I_{j,p_{j},p_{3-j}}\left(z\right)I_{3-j,p_{3-j},p_{j}}\left(z\right)}{\omega_{c,j,p_{j}}-\omega_{c,3-j,p_{3-j}}}.$$

For the simplest case $P_{1}=P_{2}=1$, this result coincides with Equation (8) under the condition of weak coupling, $|\omega_{c,1}-\omega_{c,2}|\gg \omega n_{0}^{-2}{\left|I_{12}I_{21}\right|}^{1/2}$. In the approximation considered, the absolute values of the products of coupling coefficients $I_{j,p_{j},p_{3-j}}\left(z\right)I_{3-j,p_{3-j},p_{j}}\left(z\right)$ in Equation (13) coincide with that defined previously, $I_{12}\left(z\right)I_{21}\left(z\right)$, and are independent of quantum numbers of solutions (see Equation (7)). Therefore, Equation (13) can be rewritten as follows:

(14) $$\begin{array}{l} \omega_{c}^{\left(p_{j}\right)}\left(z\right)=\omega_{c,j,p_{j}}+\Delta \omega_{p_{j}}\;\exp\left(-2\sigma \Delta d\left(z\right)\right),\\ \Delta \omega_{p_{j}}=\frac{1}{\pi {\left(n_{0}^{2}-1\right)}^{3/2}\omega_{0}}{\left(\frac{c}{r_{0}}\right)}^{3}\sum_{p_{3-j}=1}^{P_{3-j}}\frac{1}{\omega_{c,j,p_{j}}-\omega_{c,3-j,p_{3-j}}},\\ \sigma =\frac{\omega_{0}{\left(n_{0}^{2}-1\right)}^{1/2}}{c}, \end{array}$$

where $R_{eff}$ and Δd(z) are defined in Equation (3). Critically, this equation demonstrates the *universal Gaussian axial dependence* of the CFs $∼\exp\left[-\sigma {\left(R_{eff}^{-1}+\alpha^{2}{/2r}_{0}\right)z}^{2}\right]$, that is, simply expressed through the geometric parameters of the considered fiber configuration and *independent of the quantum numbers* $p_{j}$ of coupled transverse eigenstates $Q_{j,p_{j}}\left(\rho_{j}\right)$. The sum in Equation (14) depends on the values of the fiber CFs, includes both positive and negative terms, and thus, can be both positive and negative. A positive shift $\Delta \omega_{p_{j}}$ forms a CF barrier, whereas a negative shift forms a CF well. The order of magnitude of this sum can be estimated as 1/ ${\Delta \omega }_{FSR}$ or greater where ${\Delta \omega }_{FSR}$ is the characteristic free spectral range of the fiber CFs. Consequently, the preexponent is estimated as ${\Delta \omega }_{p_{j}}∼{\left(c/r_{0}\right)}^{3}{\left(\omega_{0}{\Delta \omega }_{FSR}\right)}^{-1}$. For example, setting $\omega_{0}=2\pi \cdot 200$ THz, $r_{0}=60\;\mu m$, and $n_{0}=1.45$, we find ${\Delta \omega }_{FSR}=c/\left(n_{0}r_{0}\right)=2\pi \cdot 0.55$ THz, and ${\Delta \omega }_{p_{j}}∼\pm \;2\pi \cdot 1$ GHz. Remarkably, the order of this CF variation coincides with that observed for the SNAP microresonators [17, 18, 19, 20, 21, 22, 23, 24, 25]. Rescaling ${\Delta \omega }_{p_{j}}$ to the effective radius variation ${\Delta r}_{eff}={-r}_{0}{\Delta \omega }_{p_{j}}/\omega_{0}$ commonly used in the description of SNAP microresonators, we arrive at the nanoscale ${\Delta r}_{eff}∼1$ nm or greater, characteristic for SNAP structures.

As we show in Supporting Information S1: Sections S4 and S5, even nanometer‐scale differences between fiber radii can substantially alter the structure of their WGM CF spectra and the inter‐fiber mode coupling. To demonstrate this sensitivity, in Supporting Information S1: Figure S4 we compare the spectra of silica fibers with initial radii $r_{0}=$ 62.5 μm and $\omega_{0}≅2\pi \cdot 193$ THz introducing their radius differences of only 10–30 nm. These nanoscale geometrical changes shift corresponding CFs by approximately 30–100 GHz lifting the degeneracy of modes with the same azimuthal and radial quantum numbers. In Supporting Information S1: Figure S5, we examine how individual WGMs of fiber 2 contribute to the CF shift of a selected fundamental radial WGM of fiber 1. We show that, despite the dense transverse spectrum illustrated in Supporting Information S1: Figure S4, only a limited subset of modes contributes appreciably. For identical fibers, resonant coupling between matching modes dominates at larger separations Δd(z), whereas other modes become increasingly important as the separation decreases. A small radius mismatch detunes the matching modes and increases the relative importance of these additional contributions. The increasing contribution of other modes at smaller Δd(z), together with the detuning caused by radius mismatch, limits the accuracy of the analytical approximation described by Equations (7) and (14). However, in the adiabatic approximation, which is justified for sufficiently small tilt angle α and effective fiber curvature $R_{eff}^{-1}$ or interest here, the numerical multipole method described in Supporting Information S1: Section S4 can be applied.

## 3 Experimental Considerations

Recent experimental demonstration of microresonators created at the crossing of two straight fibers showed that the variation of a tilt angle by a *milliradian* order introduces the variation of the microresonator's length by a *millimeter* order [30]. In the theoretical estimates of Ref. [30], the crossing fiber segments were assumed to be ideally straight, whereas the correspondence with the experimental data was insufficient. Here, we suggest that the observed discrepancy between theory and experiment may partly reflect neglect of very small but finite fiber curvatures. The estimates below test the plausibility of this interpretation. We show that a physically justified meter‐order‐long curvature radius in millimeter‐order‐long fibers can dramatically change the inter‐fiber coupling length and the microresonator spectrum.

Optical fiber segments, even very short ones, are never ideally straight. They can be divided into those with permanent and those with temporary curvature. Temporary curvature can be introduced mechanically or by local heating, whereas permanent curvature can be introduced by fiber melting or annealing. As an example of temporary induced curvature, Figure 6a shows a fiber segment bent by its own weight. We assume that the fiber segment has a free end and is supported at a point away from its end by distance L. Then, following the Euler–Bernoulli beam theory [38], the fiber curvature radius at the support point can be found as follows:

(15) $$R_{\text{weight}}=\frac{Er_{0}^{2}}{2\rho gL^{2}}$$

where E is the Young's modulus, ρ is the fiber density, and *g* is the acceleration due to gravity. For a silica fiber considered, we set $E=72\cdot 10^{9}$ N/m^2^, ρ=2200 kg/m^3^, *g* = 9.81 m/s^2^, and $r_{0}=62.5\;\mu m$. Then, for L=2 cm, we find $R_{\text{weight}}=16$ m and for L=5 cm, we find $R_{\text{weight}}=2.6$ m.

**FIGURE 6:**
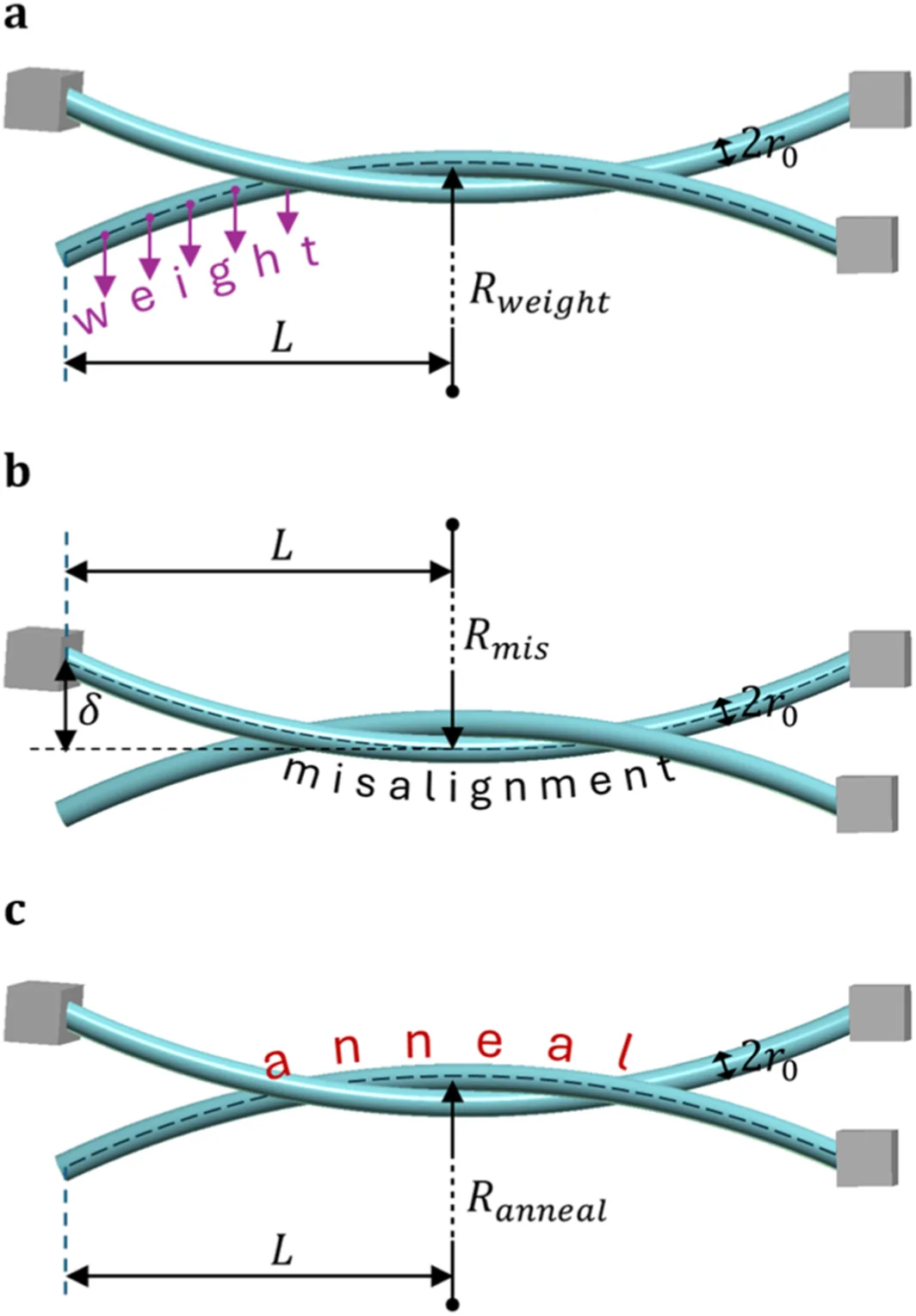
(a) A fiber segment bent by its own weight. (b) A fiber segment bent by misalignment. (c) A fiber segment bent by side annealing.

A temporary induced curvature can also be induced by improper alignment of the fiber's fixed ends, leading to a deviation δ of the fiber end from its direction at the fiber intersection point (Figure 6b). The *smallest* deviation $\delta_{\min}$ corresponds to the ideal situation in which the fiber orientation at the fiber intersection point is not altered by misalignment. The corresponding fiber curvature radius is related to this deviation as follows:

(16) $$R_{mis}=\frac{L^{2}}{3\delta_{\min}}$$

For a characteristic misalignment of translation stages $\delta_{\min}∼\;100\;\mu m$ and L∼2cm we find $R_{mis}∼1$ m.

A commonly used approach to clean the fiber surface is heating it with a CO_2_ laser or flame. This process is similar to fiber annealing and may cause asymmetric (along the fiber cross‐section) release of frozen‐in stresses, leading to permanent fiber bending. The release of residual stresses, whose characteristic value for silica fibers is $\sigma ∼10^{7}$ N/m^2^ [39, 40] leads to fiber shrinkage $\Delta \varepsilon =\sigma /E∼10^{-4}$. For side annealing, we determine the smallest possible curvature radius by modeling the fiber as a bi‐material beam composed of materials with equal widths and Young moduli, and assuming that stresses are relaxed in one material and remain unchanged in the other. Then the *smallest* induced fiber curvature radius $R_{\text{anneal}}$ can be estimated from the Timoshenko equation [41] as follows:

(17) $$R_{\text{anneal}}=\frac{4r_{0}}{3\Delta \varepsilon }=\frac{4r_{0}E}{3\sigma }∼0.6\;m.$$

It follows from the estimates based on Equations (15)‐(17) that, for different reasons, the fiber segments of the considered system can have a *meter‐order* curvature radius. These dramatically small bending effects have to be taken into account in the explanation of the results of recent experiments [30]. In Ref. [30], SNAP microresonators were created at the intersection of optical fiber segments, which were assumed to be straight, that is, to have $R_{1}=R_{2}=\infty$. The FSR of these resonators was tuned by the milliradian‐scale variation of the angle α between fibers. The microresonators were characterized using spectrograms, that is, 2D plots of their spectra measured with a transverse microfiber taper connected to the optical spectrum analyzer. The microfiber was scanned along the length of one of the fibers, periodically touching its surface at a 2 μm resolution. The spectrograms of two of these microresonators corresponding to the angles $\alpha_{1}=0.01$ rad and $\alpha_{2}=0.005$ rad are shown in Figure 7a,b, respectively. In these spectrograms, the wavelength dependence along the vertical axes used in [30] was rescaled to the frequency dependence aligned with the present paper. Generally, the coupling‐induced frequency splitting and shifting depend on the tilt angle α and effective fiber curvature radius $R_{eff}$ only through the fiber separation Δd(z) defined by Equation (3). Therefore, for ${\Delta h}_{x}=0$, we have the following equation:

(18) $$\omega_{c}^{\left(p_{j}\right)}\left(z\right)=\omega_{c,j,p_{j}}\left(\Delta d\left(z\right)\right)=\omega_{c,j,p_{j}}\left(\frac{1}{2}\left(\frac{1}{R_{eff}}+\frac{\alpha^{2}}{2r_{0}}\right)z^{2}\right).$$

**FIGURE 7:**
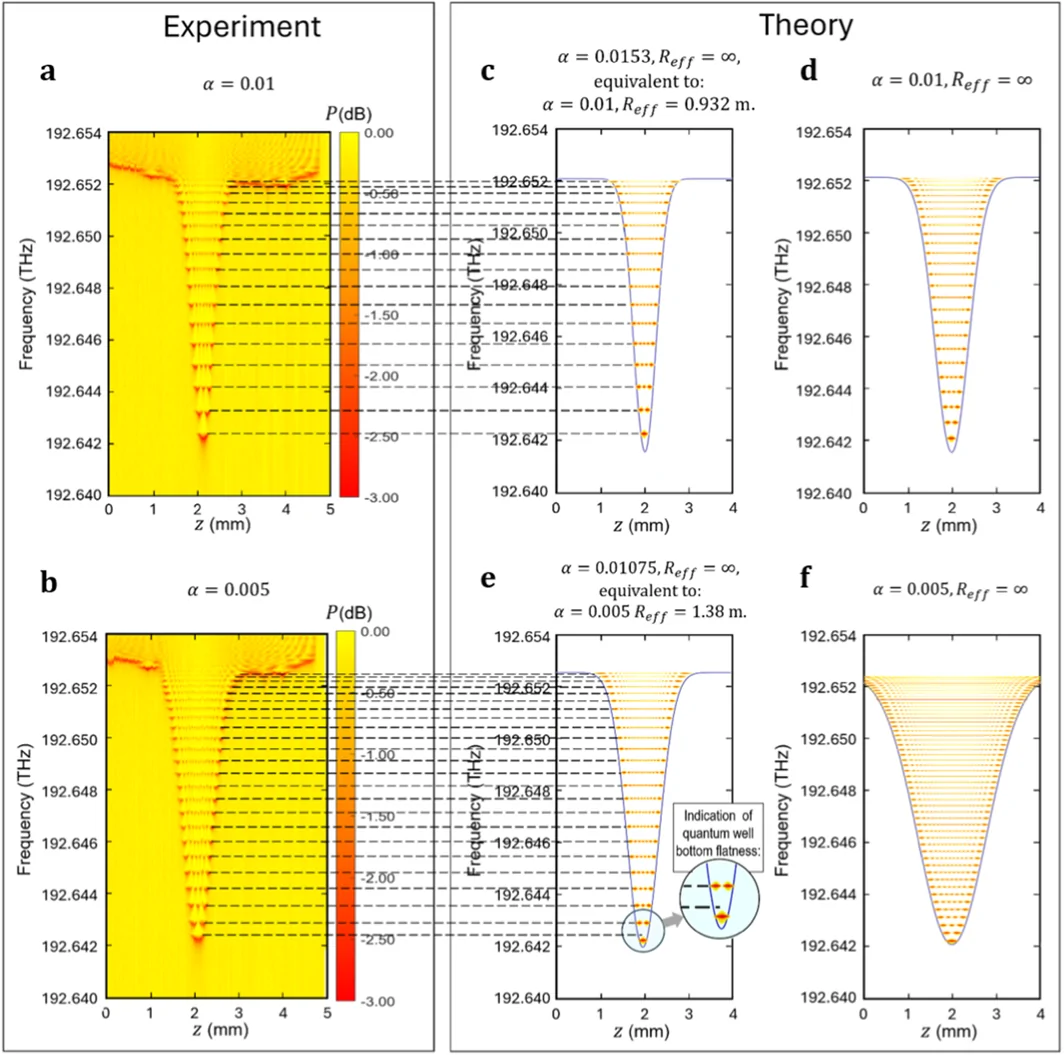
Comparison of the experimental spectrograms from Ref. [30], where the vertical wavelength coordinates are rescaled to the frequency coordinates, with theoretical CF profiles and spectra for the same inter‐fiber angles α. (a, b) Experimental spectrograms for α=0.01 and α=0.005, respectively. (c, d, e, f) The corresponding theoretical CF profiles and spectra for (c) α=0.01 and $R_{eff}=0.932$ m, (d) α=0.01 and $R_{eff}=\infty$, (e) α=0.005 and $R_{eff}=1.38$ m, and (f) α=0.005 and $R_{eff}=\infty$.

Consequently, fiber configurations having the same value of $\left(R_{eff}^{-1}+\alpha^{2}{/2r}_{0}\right)$ should exhibit identical WGM spectra.

If the fibers in Ref. [30] were straight, that is $R_{1}=R_{2}=\infty$, Equation (18) would predict that the CF profile in Figure 7b could be mapped onto that in Figure 7a by rescaling the distance from the contact point, z→χz, with the factor $\chi =\alpha_{2}/\alpha_{1}$. However, matching the measured profiles requires χ=0.74, rather than the measured angle ratio $\alpha_{2}/\alpha_{1}=0.5$. If instead we assume a finite effective fiber curvature radius $R_{eff}$ that is the same for both configurations, Equation (18) gives us the following:

(19) $$\chi ={\left(\frac{2r_{0}+\alpha_{2}^{2}R_{eff}}{2r_{0}+\alpha_{1}^{2}R_{eff}}\right)}^{1/2}$$

Using the measured scaling factor χ=0.74 and $r_{0}=62.5$ μm, Equation (19) gives $R_{eff}\approx 1.9$ m.

A quantitative numerical calculation of the CF profiles for the configuration in Ref. [30] requires the values of uncoupled CFs of the participating WGMs, which are not known with sufficient precision and are sensitive to nanoscale variations in the fiber radii (see Supporting Information S1: Figures S4 and S5). Therefore, it is also interesting to compare the experiments with the analytical models described above. For this purpose, we assume the Gaussian axial dependence $\omega_{c}\left(z\right)=\omega_{c1}+\Delta \omega \;\exp\left(-2\sigma \Delta d\left(z\right)\right)$, obtained from perturbation theory and Equation (14), and fit the spectrograms in Figure 7a,b by adjusting the effective curvature radius $R_{eff}$ and the signed CF shift Δω while retaining the experimentally measured angle α. Comparison of the experimental spectrogram in Figure 7a with the calculated CF profile and axial spectrum in Figure 7c shows good agreement for α=0.01 and $R_{eff}=0.932$ m, or, equivalently within this model, for α=0.0153 and $R_{eff}=\infty$. For comparison, Figure 7d shows the calculation for the measured angle α=0.01 with straight fibers, $R_{eff}=\infty$. The numerical examples in Supporting Information S1: Section S5 of SI indicate a weaker dependence of the CF shift on fiber separation than predicted by the perturbative planar approximation. For straight fibers, reproducing this behavior with the analytical profile would require a reduction in the effective value of α, whereas fitting the experimental spectrum requires an increase in α above its measured value. Thus, the discrepancy is not explained by this correction to the coupling approximation alone and motivates including finite fiber curvature. Similarly, the experimental spectrogram in Figure 7b, measured at α=0.005, is in reasonable agreement with the calculation in Figure 7e for $R_{eff}=1.38$ m, whereas the straight fiber calculation in Figure 7f, with $R_{eff}=\infty$, differs substantially from the experiment. The remaining discrepancy between the lowest experimental and calculated axial eigenfrequencies, highlighted by the blue circle in Figure 7e, reflects a flattening of the CF profile near the contact region due to fiber deformation under contact pressure, including that caused by electrostatic and van der Waals forces [30, 34, 35]. In Supporting Information S1: Section S6 of SI we investigate the related geometric and photoelastic effects of fiber squeezing. Our estimates show that squeezing by 1–10 nm can shift the CF by approximately 0.1–1 GHz comparable to the observed discrepancy.

## 4 Discussion

We theoretically described optical microresonators formed at the intersection of optical fibers and analyzed the physical effects underlying their shape and spectral characteristics. The CF variation of the induced microresonators is of the same order of magnitude as that in SNAP microresonators, which typically exhibit nanoscale effective radius variations along the optical fiber axis [17]. This allows us to attribute the described microresonators to the class of SNAP microresonators.

Two complementary coupling regimes have been considered. In the case of resonant interaction between closely spaced CFs, the intersecting fiber system can be described by a two‐state model. In this approximation, coupling between two adjacent CFs of the fibers leads to the well‐known effect of level repulsion [36], resulting in the formation of CF potential barriers and potential wells. At small fiber separations, we used numerical calculations to correct the analytical CF profile described by Equations (7) and (8). Because segments with sufficiently close CFs can be selected from the same parent fiber [37], the pure two‐state coupling regime could be explored experimentally at separations where contributions from other modes remain negligible. In contrast, in the most common case when the induced CF shifts are small compared to the separation of CFs, their variation can be described perturbatively, leading to the sum of small corrections accumulated from many interacting states. Then, the CF variation has a universal Gaussian shape described by Equation (14). Similar to the two‐state model, the latter approximation fails at small fiber separations when the inter‐fiber coupling becomes comparable to the CF separation. For the examples considered, the dominant contributions come from a sequence of WGMs in which increasing radial order is accompanied by decreasing azimuthal order, as illustrated in Supporting Information S1: Figure S5 of the SI. In both cases, a CF potential well localizes WGMs along the axial direction and forms a SNAP microresonator.

The comparison between the developed theory and experimentally measured spectrograms of microresonators induced at the intersection of optical fibers [30] showed that inclusion of the dramatically small but unavoidable curvature of the fiber segments is essential for achieving quantitative agreement with the measured spectra. Whereas models neglecting curvature reproduce only the qualitative features of coupling‐induced localization, they fail to account for the observed CF profiles and eigenfrequency distributions. By incorporating the meter‐scale effective fiber curvature radius into the geometrical description of the fibers and the resulting coupling coefficients, the developed theory allows to explain the experimentally observed CF frequency variation and spectral characteristics of the induced microresonators.

It is also interesting to investigate losses of microresonator eigenstates induced by coupling to other fiber modes, as well as nonadiabatic effects at larger tilt angles or smaller curvature radii, where the coupling coefficients vary more rapidly along the axial coordinate z. Future studies may also address more complex configurations involving the intersection of fibers with pre‐inscribed SNAP microresonator structures, as well as multiple intersecting fibers, thereby opening new opportunities for realizing SNAP devices with enhanced tunability and reconfigurability.

## Author Contributions

**M. Sumetsky:** conceptualization, data curation, formal analysis, visualization, writing–original draft, methodology, investigation, funding acquisition, validation, writing–review and editing, software, resources.

## Funding

This work was supported by the Engineering and Physical Sciences Research Council (EPSRC) (Grant Nos. EP/X03772X/1 and EP/W002868/1).

## Conflicts of Interest

The author declares no conflicts of interest.

## Supporting information


Supporting Information S1

## Data Availability

The data that support the findings of this study are available from the corresponding author upon reasonable request.
