# Supplementary material for: Widely Tunable SNAP Microresonators Induced at the Optical Fiber Intersections
Source: Nanophotonics. 2026 Nov 13;15(22):e70366. doi: 10.1002/nap2.70366 (PMC13764582; doi:10.1002/nap2.70366)
Supplement: Supplementary file 1 [file NAP2-15-e70366-s001.pdf]

SUPPORTING INFORMATION

Widely tunable SNAP microresonators induced at the optical fiber intersections

M. Sumetsky
Aston Institute of Photonic Technologies, Aston University, Birmingham B4 7ET, UK
m.sumetsky@aston.ac.uk

S1. WGMS in a single fiber

Here we consider WGMs of uncoupled fiber. Separating variables in local cylindrical coordinates (ρ, θ, z), we define the solution of the wave equation as $E_{m,q}(\mathbf{r}) = \exp(i\beta(\omega)z)\exp(im\theta)Q_q(\rho)$, where $Q_q(\rho)$ satisfies the equation [S1]

$$\frac{d^2Q}{d\rho^2}+\frac{1}{\rho}\frac{dQ}{d\rho}+\left(\frac{\omega^2}{c^2}n^2(\rho)-\frac{m^2}{\rho^2}\right)Q=0\,, \tag{S1}$$

corresponding to cutoff frequencies, for which $\beta(\omega) = 0$. For WGMs of our concern, we are only interested in fundamental and close to fundamental eigenstates of this equation having quantum numbers $q = 1,2, ...$. These states are localized near the fiber surface, $\rho = r_0$, where Eq. (S1) is simplified to the equation:

$$\frac{d^2Q}{dx^2}+\frac{1}{r_0}\frac{dQ}{dx}+\left(\frac{\omega^2}{c^2}n^2(x)-\frac{m^2}{r_0^2}+\frac{2m^2}{r_0^3}x\right)Q=0\,, \tag{S2}$$

where $x = \rho - r_0$ and

$$n(x)=\begin{cases} n_0, & x<0 \\ 1, & x>0 \end{cases} \tag{S3}$$

Solution of this equation in the region $\rho < r_0$ is expressed through the Airy function:

$$Q_q(\rho)=A_q\cdot \mathrm{Ai}\left(\varsigma(r_0-\rho)+a_q\right),\quad \varsigma=\frac{(2m^2)^{1/3}}{r_0}\cong\left(\frac{2n_0^2\omega^2}{c^2r_0}\right)^{1/3}, \tag{S4}$$

$$a_1=-2.338,\quad a_2=-4.088,\quad a_3=-5.521,\quad a_q \underset{q\gg 1}{\simeq} -\left(\frac{3\pi}{2}\left(q-\frac{1}{4}\right)\right)^{2/3}, \tag{S5}$$

where a_q are the roots of Airy function and we assumed that $Q_q(r_0) = 0$, which is known to be in good agreement with numerical modelling [S2]. Remarkably, the asymptotic expression for the roots a_q in Eq. (S5) is accurate with better than 1% starting from $q = 1$. Using this asymptotic, the normalization of the solution in the fiber cross-section $U(\rho, \theta) = \exp(im\theta)Q_q(\rho)$, which is reduced to the condition $2\pi r_0 \int_{-\infty}^{r_0} d\rho Q_q^2(\rho) = 1$, yields:

$$A_q=\left(\frac{\varsigma}{2r_0}\right)^{1/2}\left[\frac{3\pi}{2}\left(q-\frac{1}{4}\right)\right]^{-1/6}. \tag{S6}$$

Similar to the asymptotic for a_q from Eq. (S5), this asymptotics is accurate with better than 1% starting with $q = 1$.

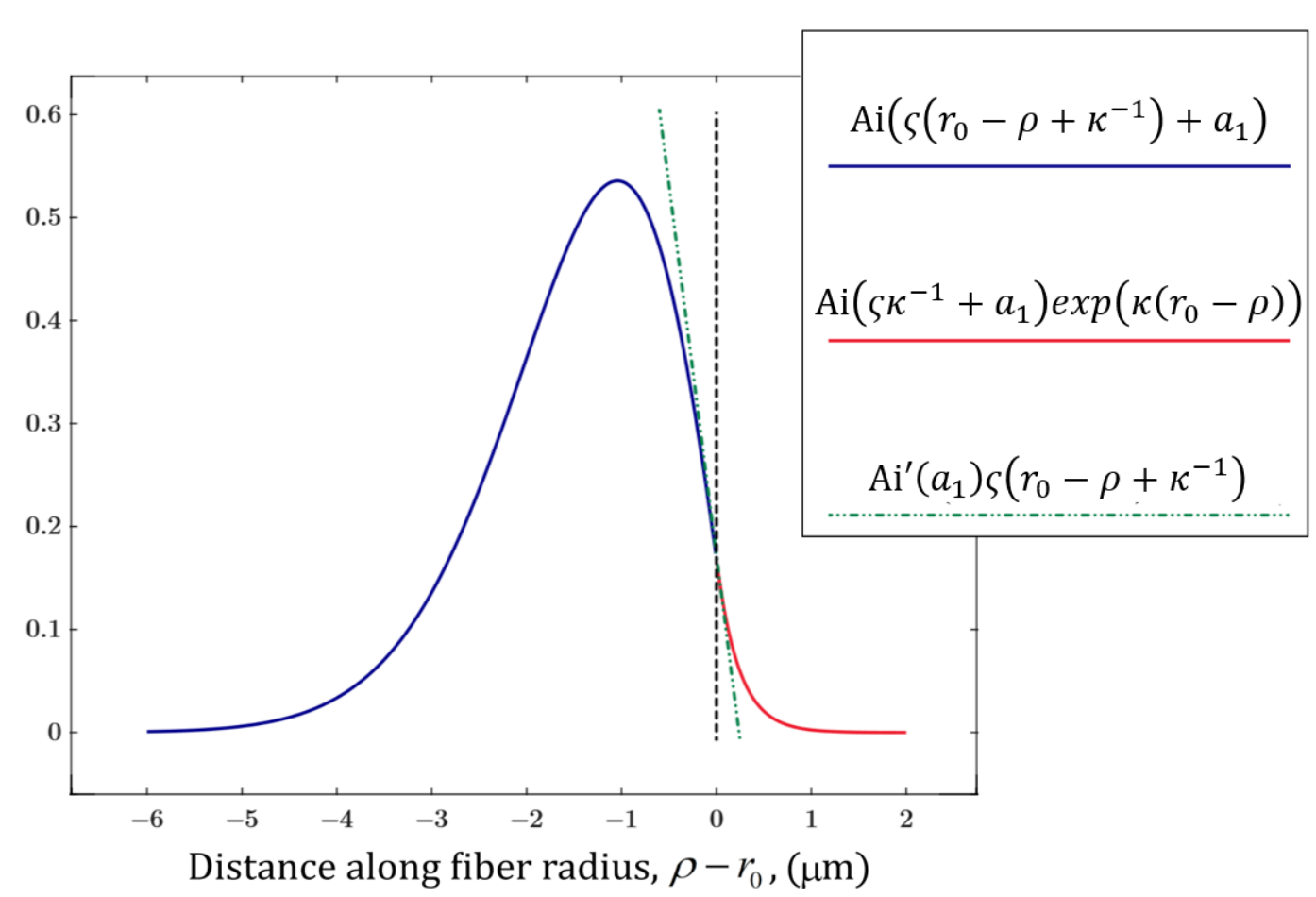

Fig. S1. Distribution of the fundamental WGM along the fiber radial direction. The distribution inside the fiber, expressed through the Airy function (blue), is matched with the exponential distribution outside the fiber (red).

Next, we abandon the assumption $Q_q(r_0) = 0$ and match the solution of Eq. (S1) inside the fiber with the evanescent solution at $\rho > r_0$ (Fig. S1). In this case, the solution near the fiber surface, $\rho = r_0$, is slightly shifted from zero and can be presented in the form:

$$Q_q(\rho) = A_q \cdot \mathrm{Ai}\left(\varsigma\left(r_0 - \rho\right) + a_q + \Delta a_q\right) \tag{S7}$$

with the same normalization constant A_q defined by Eq. (S6). Assuming that the solution considered is a TE mode, we match this solution and its derivative at $\rho = r_0$ with the evanescent solution at $\rho > r_0$,

$$Q_q(\rho) = B_q \exp\left(-\kappa\left(\rho - r_0\right)\right), \quad \kappa = \left(\frac{m^2}{r_0^2} - \frac{\omega^2}{c^2}\right)^{1/2} = \frac{\omega}{c}\left(n_0^2 - 1\right)^{1/2}, \tag{S8}$$

leads to the equation for Δa_q and expression for B_q:

$$\frac{\mathrm{Ai}'\left(a_q + \Delta a_q\right)}{\mathrm{Ai}\left(a_q + \Delta a_q\right)} = \frac{\kappa}{\varsigma}, \quad B_q = A_q \mathrm{Ai}\left(a_q + \Delta a_q\right). \tag{S9}$$

Assuming $\Delta a_q \ll 1$, we find:

$$\begin{gathered}\mathrm{Ai}(a_q + \Delta a_q) \cong \mathrm{Ai}'(a_q)\Delta a_q, \\ \mathrm{Ai}'(a_q) \cong (-1)^{q-1}\pi^{-1/2}\left[\frac{3\pi}{2}\left(q - \frac{1}{4}\right)\right]^{1/6}\end{gathered} \tag{S10}$$

and Eq. (S9) is simplified:

$$\Delta a_q = \frac{\varsigma}{\kappa}, \quad B_q = (-1)^{q-1}\left(\frac{\varsigma^3}{2\pi r_0}\right)^{1/2}\frac{1}{\kappa}. \tag{S11}$$

From Eqs. (S4) and (S8)-(S11), we find for the evanescent field at $\rho > r_0$:

$$Q_q(\rho) = \frac{(-1)^{q-1} n_0}{\pi^{1/2}\left(n_0^2 - 1\right)^{1/2} r_0}\exp\left(-\kappa\left(\rho - r_0\right)\right). \tag{S12}$$

Remarkably, the magnitude of this field is *independent of the radial quantum number* q.

From Eqs. (S4), (S6), and (S10)-(S12), we find inside the fiber near its surface:

$$\begin{aligned}Q_q(\rho) &= A_q \cdot \mathrm{Ai}\left(\varsigma\left(r_0 - \rho\right) + a_q + \Delta a_q\right) \cong A_q \cdot \mathrm{Ai}'\left(a_q\right)\left(\varsigma\left(r_0 - \rho\right) + \Delta a_q\right) \\ &\cong (-1)^{q-1}\pi^{-1/2}\frac{n_0\omega_0}{c r_0}\left(\left(r_0 - \rho\right) + \frac{1}{\kappa}\right)\end{aligned} \tag{S13}$$

S2. Coupled wave equations

The WGMs $E(\mathbf{r})$, which propagate along the configuration of fibers illustrated in Fig. 2, satisfy the wave equation, Eq. (4), of the main text. For each of the fibers, it is convenient to use the cylindrical curvilinear coordinates (ρ_j, θ_j, s_j), where s_j coincides with the axis of the fiber j. Then, for a small deformation assumed and close to the fiber contact point corresponding to $s_1 = s_2 = 0$, we have

$$\begin{aligned}\rho_2 &= 2r_0 + \Delta h_x - \rho_1 + \frac{1}{2}\left(\frac{1}{R_2} - \frac{1}{R_1}\right)z^2 + \frac{\alpha^2 z^2}{2r_0} + r_0\theta_1^2 - \alpha\theta_1 z \\ \theta_2 &= \pi - \theta_1 + \frac{\alpha z}{r_0} \\ s_2 &= s_1 = z\end{aligned} \tag{S14}$$

In the approximation considered, the coordinates along the fiber axes s_j coincide with the coordinate along the straight axis z (see Fig. 2). Assuming first that each of the fibers stands alone, we can write down the WGM solution for the fiber j as $E_j^{(0)}(\mathbf{r}) = \exp(i\beta_j(\omega)z)U_j(\rho_j, \theta_j)$ where the transverse component $U_j(\rho_j, \theta_j) = \exp(im_j\theta_j)Q_{jq}(\rho_j)$ satisfies the 2D wave equation [S1]

$$\Delta_{T_j} U_j(\rho_j, \theta_j) + \left(\frac{\omega^2}{c^2} n_j^2(\rho_j, \theta_j) - \beta_j^2(\omega)\right)U_j(\rho_j, \theta_j) = 0. \tag{S15}$$

Here $n_j(\rho_j, \theta_j)$ is the refractive index distribution of the fiber j standing alone, and the dependence of $\beta_j(\omega)$ on the angular frequency of light ω is determined from the condition of vanishing of $Q_{jq}(\rho_j)$ at large ρ_j. For the assumed close proximity to the CFs $\omega_{c,1}$ and $\omega_{c,2}$, we have [S3]

$$\beta_j^2(\omega) = 2\omega_0 \frac{n_0^2}{c^2}\left(\omega - \omega_{c,j}\right). \tag{S16}$$

Here $\omega_0 \cong \omega_{cj}$ is the frequency in the narrow bandwidth considered.

We assume that the WGM CFs of fiber 1 and fiber 2, $\omega_{c,1}$ and $\omega_{c,2}$, are close to each other and to the frequency of light ω and separated from other CFs, so that we can ignore the effect of coupling with other transverse states. Then, the solution of the wave equation, Eq. (4), can be found in the form

$$E(\mathbf{r}) = \Psi_1(z)U_1(\rho_1,\theta_1) + \Psi_2(z)U_2(\rho_2,\theta_2). \tag{S17}$$

In the calculations below, we assume that $U_j(\rho_j,\theta_j) = \exp(im\theta_j)Q_{jq}(\rho_j)$ where the radial component $Q_{jq}(\rho_j)$ is defined in Section S1.

Substituting Eq. (S17) into Eq. (4) and using Eq. (S15) for $j = 1$ and 2, we find:

$$\begin{aligned}&\left[\beta_1^2(\omega) + k_0^2\left(n^2(\mathbf{r}) - n_1^2(\rho_1,\theta_1)\right)\right]U_1(\rho_1,\theta_1)\Psi_1(z) + \\ &\left[\beta_2^2(\omega) + k_0^2\left(n^2(\mathbf{r}) - n_2^2(\rho_2,\theta_2)\right)\right]U_2(\rho_2,\theta_2)\Psi_2(z) + \\ &U_1(\rho_1,\theta_1)\frac{d^2\Psi_1(z)}{dz^2} + U_2(\rho_2,\theta_2)\frac{d^2\Psi_2(z)}{dz^2} = 0\end{aligned} \tag{S18}$$

Next, using Eq. (S14) we express U_2 in Eq. (S18) through coordinates (ρ_1, θ_1, z) as $U_2(\rho_2(\rho_1, \theta_1 z), \theta_2(\rho_1, \theta_1, z))$. Multiplying Eq. (S18) by $U_1^*(\rho_1, \theta_1)$ and integrating over the plane (ρ_1, θ_1) and, similarly, multiply it by $U_2^*(\rho_2, \theta_2) = U_2^*(\rho_2(\rho_1, \theta_1, z), \theta_2(\rho_1, \theta_1, z))$ and integrating over the plane (ρ_1, θ_1), we arrive at the coupled wave equations:

$$\begin{aligned}\frac{d^2\Psi_1}{dz^2} + \beta_1^2(\omega)\Psi_1 &= -\frac{\omega_0^2}{c^2}I_{12}(z)\Psi_2 \\ \frac{d^2\Psi_2}{dz^2} + \beta_2^2(\omega)\Psi_2 &= -\frac{\omega_0^2}{c^2}I_{21}(z)\Psi_1\end{aligned} \tag{S19}$$

In these equations, for the assumed small bending and tilting of fibers (see Eqs. (S14)), we introduced the dimensionless coupling parameters:

$$I_{12}(z) = I_{21}^* = \left(n_0^2 - 1\right)\int_0^{r_0}\int_{\theta_1} Q_1(\rho_1,\theta_1)Q_2^*(\rho_2(\rho_1,\theta_1,z),\theta_2(\rho_1,\theta_1,z))\rho_1 d\theta_1 d\rho_1 \tag{S20}$$

where $Q_j(q_j, \theta_j, z)$ are the normalized solutions defined in Section S1. Using Eq. (S15) for $j = 1{,}2$ and for the case $\beta_j(\omega) \ll n_j\omega/c$ of our interest (see Eq. (S16)), the integral over the cross-section of fiber 1 in Eq. (S20) can be transformed to the integral along the axis $\ell_\perp$ normal to the axis v connecting the fiber centers as shown in Fig. S2:

$$I_{12}(z) = I_{21}^*(z) = \frac{c^2}{\omega^2}\int_{\ell_\perp}\left(Q_1^*\frac{\partial Q_2}{\partial v} - Q_2^*\frac{\partial Q_1}{\partial v}\right)d\ell_\perp. \tag{S21}$$

To calculate this integral, we assume that the WGMs in fibers have the same azimuthal quantum numbers m_j and radial quantum numbers q_j choose the axis $\ell_\perp$ at equal distances from the fibers where $Q_2 = Q_1$ and $\partial Q_2/\partial v = -\partial Q_1/\partial v$. Then, using Eq. (S12) for the normalized solution Q_j we arrive at Eq. (7) of the main text.

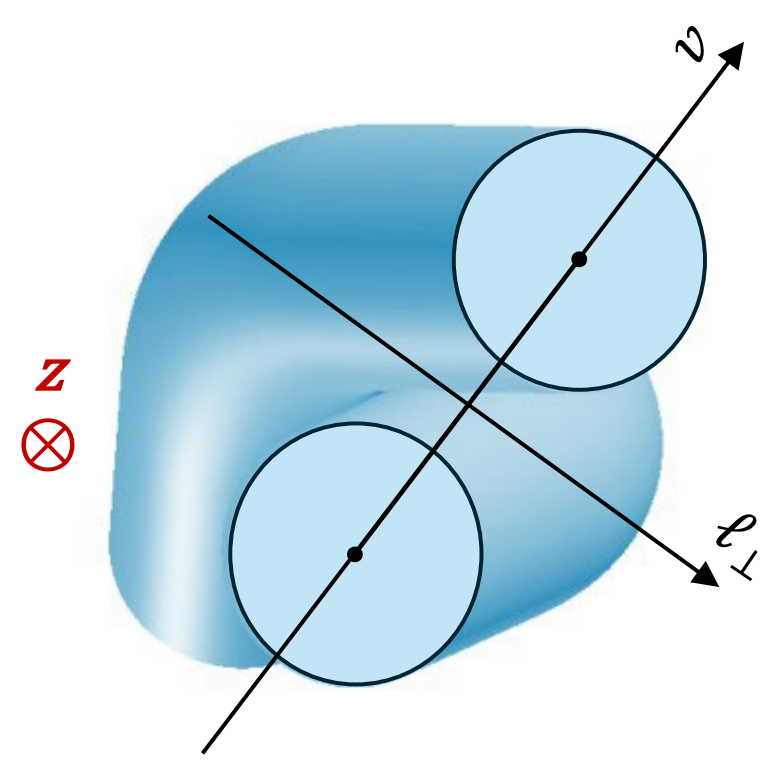

Fig. S2. Integration axis $l_\perp$ in Eq. (S21).

Coupled wave equations, Eqs. (S19), can be reduced to the fourth-order equation for $\Psi_1(z)$:

$$\frac{d^4\Psi_1}{dz^4}+a_3(z)\frac{d^3\Psi_1}{dz^3}+a_2(z)\frac{d^2\Psi_1}{dz^2}+a_1(z)\frac{d\Psi_1}{dz}+a_0(z)\Psi_1=0\,, \tag{S22}$$

where

$$\begin{aligned}
a_0(z,\omega)&=\beta_1^2(\omega)\left[\beta_2^2(\omega)+2\left(\frac{1}{I_{12}(z)}\frac{dI_{12}(z)}{dz}\right)^2-\frac{1}{I_{12}(z)}\frac{d^2I_{12}(z)}{dz^2}\right]-\frac{\omega_0^4}{c^4}I_{12}(z)I_{21}(z)\\
a_1(z,\omega)&=-2\frac{\beta_1^2(\omega)}{I_{12}(z)}\frac{dI_{12}(z)}{dz}\\
a_2(z,\omega)&=\beta_1^2(\omega)+\beta_2^2(\omega)+2\left(\frac{1}{I_{12}(z)}\frac{dI_{12}(z)}{dz}\right)^2-\frac{1}{I_{12}(z)}\frac{d^2I_{12}(z)}{dz^2}\\
a_3(z,\omega)&=-\frac{2}{I_{12}(z)}\frac{dI_{12}(z)}{dz}
\end{aligned} \tag{S23}$$

S3. WKB and adiabatic solution of coupled wave equations

Assuming that the coefficients in Eqs. (S22) are sufficiently slow functions of z, we look for the solution of this equation in the WKB approximation. For this purpose, we formally add the factors ε^n in front of nth derivatives in Eqs. (S22) and (S23) and look for the solution of Eq. (S22) in the form

$$\Psi_1(z)=\left(A_0(z)+\varepsilon A_1(z)+...\right)\exp\left(\frac{i}{\varepsilon}\int^z\beta(z,\omega)dz\right) \tag{S24}$$

In the zero and first orders in ε, we arrive at the quartic algebraic equation for the propagation constant $\beta(z,\omega)$,

$$\beta^4(z,\omega)-\left(\beta_1^2(\omega)+\beta_2^2(\omega)\right)\beta^2(z,\omega)+\beta_1^2(\omega)\beta_2^2(\omega)-k_0^4I_{12}(z)I_{21}(z)=0 \tag{S25}$$

and the first-order linear differential equation for the preexponent $A_0(z,\omega)$,

$$\frac{dA_0}{dz}+P(z,\omega)A_0=0, \tag{S26}$$

$$P(z,\omega)=\frac{\left(a_{20}(z,\omega)-6\beta^2(z,\omega)\right)\dfrac{d\beta(z,\omega)}{dz}+a_1(z,\omega)\beta(z,\omega)-a_3(z,\omega)\beta^3(z,\omega)}{2\beta(z,\omega)\left(a_{20}(z,\omega)-2\beta^2(z,\omega)\right)}. \tag{S27}$$

where

$$\begin{aligned}
a_{00}(z,\omega)&=\beta_1^2(\omega)\beta_2^2(\omega)-k_0^4I_{12}(z)I_{21}(z)\\
a_1(z,\omega)&=2\beta_1^2(\omega)\left[\mu(\omega)z-i\beta_0\right]\\
a_{20}(z,\omega)&=\beta_1^2(\omega)+\beta_2^2(\omega)\\
a_3(z,\omega)&=2\mu(\omega)z-2i\beta_0\\
\mu(\omega)&=\frac{\omega(n_0^2-1)^{1/2}}{c}\left(\frac{1}{R_{eff}}+\frac{\alpha^2}{2r_0}\right)
\end{aligned} \tag{S28}$$

The propagation constants of the coupled fiber system $\beta(z,\omega)=\beta_\pm(z,\omega)$ are found from Eq. (S25) as:

$$\beta_\pm^2(z,\omega)=\frac{1}{2}\left(\beta_1^2(\omega)+\beta_2^2(\omega)\right)\pm\sqrt{\frac{1}{4}\left(\beta_1^2(\omega)-\beta_2^2(\omega)\right)^2+\frac{\omega_0^4}{c^4}I_{12}(z)I_{21}(z)} \tag{S29}$$

Substituting the dependencies of $\beta_j(\omega)$ from Eq. (S16), we rewrite Eq. (S29) as

$$\beta_\pm^2(z,\omega)=\omega_0\frac{n_0^2}{c^2}\left(\left(2\omega-\omega_{c1}-\omega_{c2}\right)\pm\sqrt{\left(\omega_{c1}-\omega_{c2}\right)^2+\frac{\omega_0^2}{n_0^4}I_{12}(z)I_{21}(z)}\right) \tag{S30}$$

The CFs $\omega_c^\pm(z)$ of the system considered given by Eq. (8) of the main text can be simply found from this equation by setting $\beta_\pm^2(z)=0$.

Since the coefficient $P(z,\omega)$ in Eq. (S26) for the pre-exponent $A_0(z)$ is invariant under the change of the sign of the propagation constant $\beta(z,\omega)$, pre-exponent $A_0(z)$ is invariant under this change as well. Consequently, the eigenmodes of a SNAP resonator confined by the induced CF variations $\omega_c^{\pm}(z)$ can be constructed as a linear combination of the WKB solutions $A_0(z)\sum_{\pm} exp\left(\pm i \int^z \beta(z,\omega)dz\right)$ and, in particular, presented in the form

$$A_0(z)\cos\left(\int_{z_1}^{z}\beta_{\pm}(z,\omega)ds+\varphi_0\right). \tag{S31}$$

where the phase φ_0 is determined by matching this solution with exponentially decaying solutions away from the turning points (zeros of the propagation constant $\beta(z,\omega)$) z_1 and z_2 [S4].

In the adiabatic approximation, we ignore all derivatives in Eqs. (S23), i.e., set

$$\begin{aligned} a_0(z,\omega) &= \beta_1^2(\omega)\beta_2^2(\omega)-\frac{\omega_0^4}{c^4}I_{12}(z)I_{21}(z) \\ a_1(z,\omega) &= 0 \\ a_2(z,\omega) &= \beta_1^2(\omega)+\beta_2^2(\omega) \\ a_3(z,\omega) &= 0 \end{aligned} \tag{S32}$$

and simplify the expression for $P(z,\omega)$ in Eq. (S27) to

$$P(z,\omega)=\frac{\left(\beta_1^2(\omega)+\beta_2^2(\omega)-6\beta^2(z,\omega)\right)\dfrac{d\beta(z,\omega)}{dz}}{2\beta(z,\omega)\left(\beta_1^2(\omega)+\beta_2^2(\omega)-2\beta^2(z,\omega)\right)}. \tag{S33}$$

Then, the solution of Eq. (S26) is

$$A_0(z,\omega)=\frac{A_{00}}{\sqrt{\beta(z,\omega)\left(\beta_1^2(\omega)+\beta_2^2(\omega)-2\beta^2(z,\omega)\right)}}. \tag{S34}$$

where $\beta(z,\omega)=\beta_{\pm}(z,\omega)$ is determined by Eqs. (S30), or, equivalently, by Eqs. (8) and (9) of the main text.

S4. Multipole eigenfrequency calculation method

In the adiabatic approximation, at a fixed surface gap $\Delta d(z)=\Delta d_0$, the cutoff frequencies of the coupled fibers are obtained as eigenfrequencies of the two-dimensional transverse problem (Fig. S3). Here we assume that the fiber radii r_{01}, r_{02} and refractive indices n_1, n_2 may be different. The calculation considers each local cross-section as two circular, axially uniform dielectric cylinders. Contact deformation and photoelastic changes are considered separately in section S6.

For an isolated fiber $j=1,2$, continuity of the axial TE electric field and its derivative along the fiber j radial coordinate r_j at $r_j=r_{0j}$ gives [S1]

$$n_j\frac{J_{m_j}'(n_j s_j)}{J_{m_j}(n_j s_j)}=\frac{H_{m_j}^{(1)\prime}(s_j)}{H_{m_j}^{(1)}(s_j)},\quad s_j=k(\omega)r_{0j},\quad k(\omega)=\frac{\omega}{c} \tag{S35}$$

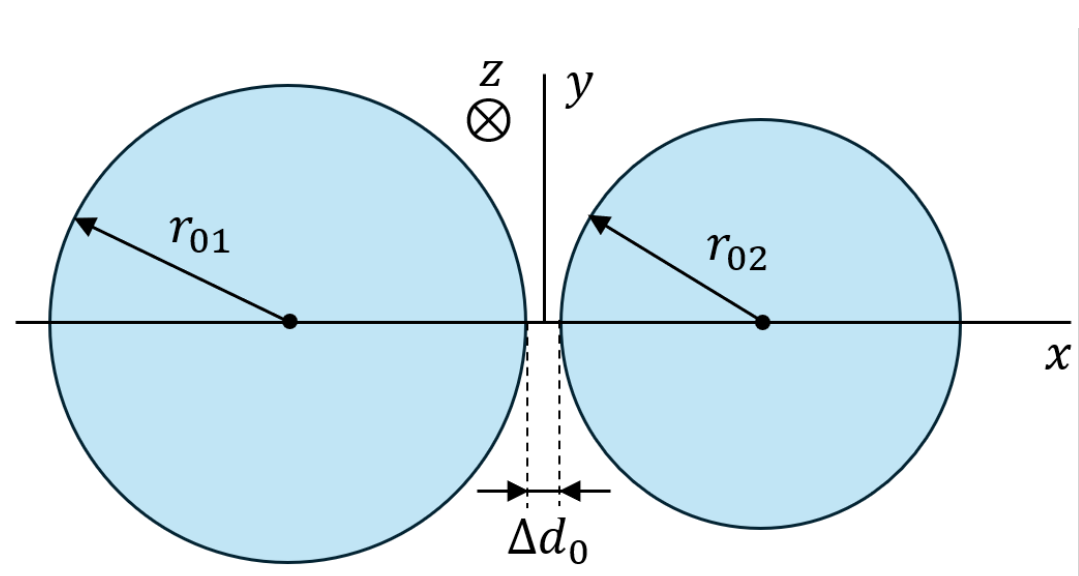

Fig. S3. Cross-section of fibers with radii r_{01} and r_{02} separated by a gap Δd_0.

Here J_m and $H_m^{(1)}$ are the Bessel and outgoing Hankel functions, m_j is the positive azimuthal order selected on fiber j, and the prime denotes the derivative of the entire argument.

For a system of two fibers, the multipole eigenfrequency calculation method (see e.g., [S5, S6]) used in our calculation is described as follows. We place two fibers side by side, with their axes separated by $d = r_{01} + r_{02} + \Delta d_0$. We expand the exterior field as outgoing cylindrical waves about both fiber axes and each interior field as regular Bessel waves about its own axis. Graf's addition theorem expresses the field outgoing from cylinder k as regular harmonics incident on cylinder $j \neq k$ [S5-S7]. We define

$$\Lambda_{i,j\ell} = n_j \frac{J_\ell'\left(n_j s_j\right)}{J_\ell\left(n_j s_j\right)}, \quad \Lambda_{J,j\ell} = \frac{J_\ell'\left(s_j\right)}{J_\ell\left(s_j\right)}, \quad \Lambda_{H,j\ell} = \frac{H_\ell^{(1)'}\left(s_j\right)}{H_\ell^{(1)}\left(s_j\right)}, \quad D_{j\ell} = \frac{\Lambda_{H,j\ell} - \Lambda_{i,j\ell}}{\Lambda_{i,j\ell} - \Lambda_{J,j\ell}}, \tag{S36}$$

and

$$T_{jk,\ell\ell'}^{(h)} = \frac{J_\ell\left(s_j\right)}{H_\ell^{(1)}\left(s_j\right)}\left[H_{\ell+\ell'}^{(1)}\left(k_0 d\right) + h\left(-1\right)^\ell H_{\ell-\ell'}^{(1)}\left(k_0 d\right)\right]. \tag{S37}$$

Here, the row number ℓ belongs to fiber j and the column number ℓ' to fiber k, and $h = \pm 1$ defines the parity of solution along axis y. We consider the modes with quantum numbers within $\mathcal{L}_j = \{m_j - L, \ldots, m_j + L\}$ on each fiber. Then, introducing $\mathbf{D}_j = \mathrm{diag}\{D_{j\ell}: \ell \in \mathcal{L}_j\}$ and the blocks $\mathbf{T}_{jk}^{(h)}$, we arrive at the eigenfrequency equation:

$$\det\begin{pmatrix} \mathbf{D}_1 & -\mathbf{T}_{12}^{(h)} \\ -\mathbf{T}_{21}^{(h)} & \mathbf{D}_2 \end{pmatrix} = 0. \tag{S38}$$

We solve this eigenfrequency equation numerically for a range of fibre gaps Δd_0. Starting at a sufficiently large gap, where the solutions approach the isolated-fibre eigenfrequencies defined by Eq. (S35), we track the roots as the gap is reduced.

Standard silica optical fibres have a dense spectrum of WGM eigenfrequencies in the frequency range of our interest. Figure S4 shows the isolated-fiber eigenfrequencies between 192 and 193.5 THz, including the mode with $m = 353$ and $q = 1$ used in our numerical comparisons. The refractive index of both fibers is $n_0 = 1.44$; the radii are $r_{01} = 62.5$ µm and $r_{02} = r_{01} + \Delta r$, with $\Delta r = 0{,}10{,}20{,}30$ nm. Even these small radius differences produce appreciable shifts of the eigenfrequencies, which are defined by rescaling $\Delta\omega/\omega_0 = -\Delta r/r_{01}$.

Among the many modes of fibers 1 and 2 in the vicinity of the mode with $m = 353$ and $q = 1$ shown in Fig. S4, only a relatively small subset contributes appreciably to the coupling-induced eigenfrequency splitting for equal fibers and to the eigenfrequency shift for unequal fibers as the gap Δd_0 is varied. Figure S5 shows, on a logarithmic scale, the magnitudes $|\Delta\omega|/(2\pi)$ of the frequency shifts of the $(m_1, q_1) = (353{,}1)$ mode of fiber 1 induced by coupling to individual modes (m_2, q_2) of fiber 2. The dominant contributions lie near the frequency-matching curve $\omega_{m_2,q_2}^{(2)} \simeq \omega_{353,1}^{(1)}$. For unequal fibers, the number of modes of fiber 2 that contribute appreciably decreases only slightly as Δd_0 increases. For equal fibers, however, the contribution of the matching mode, $m_2 = m_1 = 353$ and $q_2 = q_1 = 1$, exceeds those of the other individual modes by factors of approximately 80, 14, 7 and 4 at $\Delta d_0 = 1$, 0.5, 0.3 and 0.1 µm, respectively. The next section S5 discusses the two-mode approximation, which follows from the equations presented here by retaining only the coupling between modes with $m_2 = m_1$ and $q_2 = q_1$.

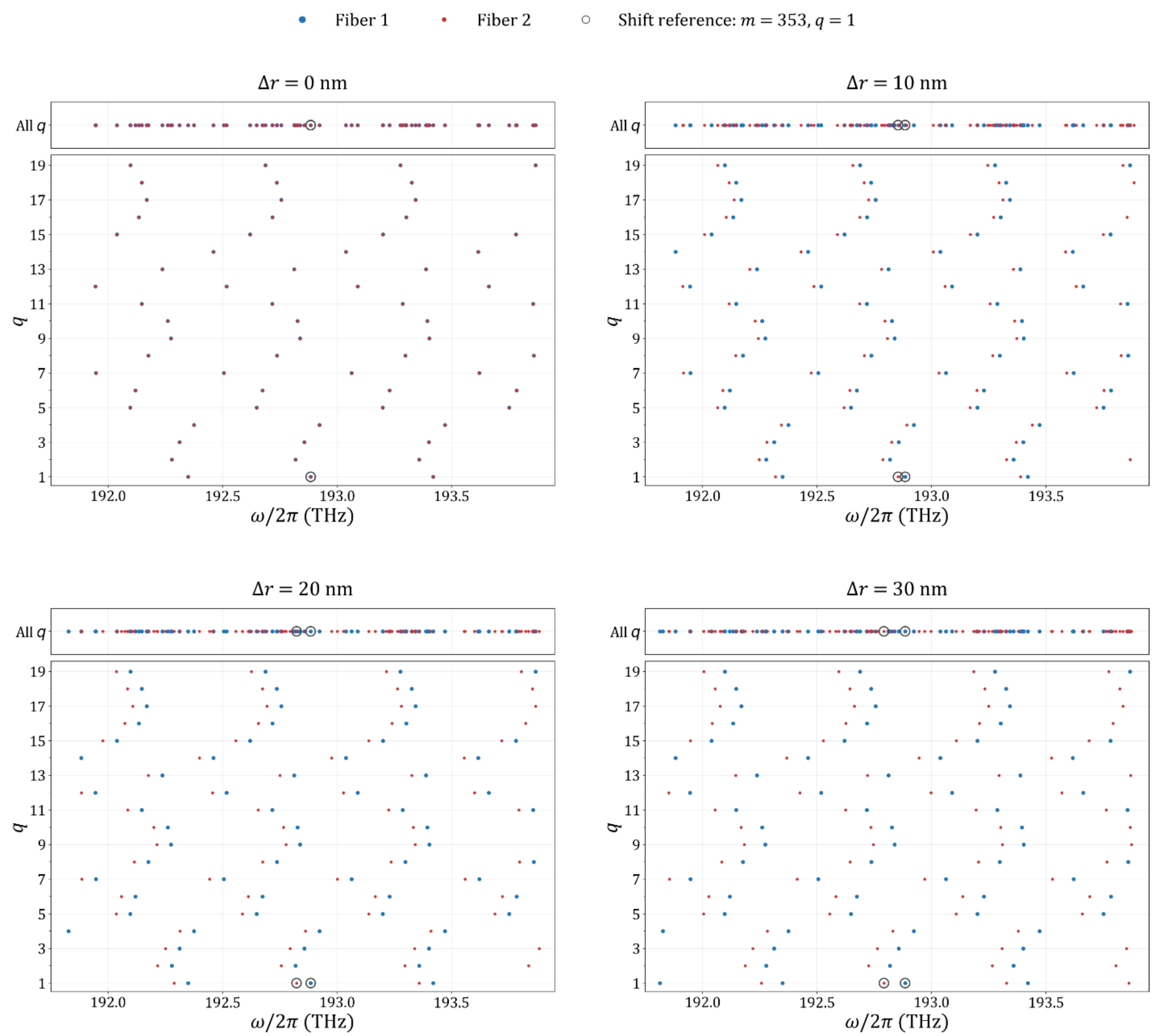

Fig. S4. The eigenfrequencies of isolated fibers in the bandwidth between 192 and 193.5 THz, including the mode with $m = 353$ and $q = 1$.

S5. Cylindrical and planar weak coupling approximation, comparison with numerical calculations

We assume now that fibers 1 and 2 have close isolated eigenfrequencies, $\omega_{m_1}^{(1)}$ and $\omega_{m_2}^{(2)}$ with $q_2 = q_1 = 1$ and retain only one mode with quantum number m_j in fiber j. Then Eq. (S38) is reduced to

$$D_{1m_1} D_{2m_2} - T_{12,m_1 m_2}^{(h)} T_{21,m_2 m_1}^{(h)} = 0 \tag{S39}$$

Using $H_{-q}^{(1)} = (-1)^q H_q^{(1)}$, setting $\omega_{m_1 m_2}^{(c)} = (\omega_{m_1}^{(1)} + \omega_{m_2}^{(2)})/2$, and linearizing D_{jm_j} in frequency near $\omega_{m_j}^{(j)}$, we define the coupling K_h in frequency units from the equation

$$K_h^2(\Delta d_0) = \frac{T_{12,m_1 m_2}^{(h)}\left(\omega_{m_1 m_2}^{(c)}, \Delta d_0\right) T_{21,m_2 m_1}^{(h)}\left(\omega_{m_1 m_2}^{(c)}, \Delta d_0\right)}{D_{1m_1}'\left(\omega_{m_1}^{(1)}\right) D_{2m_2}'\left(\omega_{m_2}^{(2)}\right)}, \tag{S40}$$

where

$$D_{jm_j}{}'(\omega_j) = \frac{dD_{jm_j}}{d\omega} = \frac{\pi s_{j0}^2 \left(n_j^2 - 1\right) J_{m_j}\left(s_{j0}\right) H_{m_j}^{(1)}\left(s_{j0}\right)}{2i\omega_{m_j}^{(j)}}, \quad s_{j0} = \frac{\omega_j r_{0j}}{c}.$$

The roots of the reduced equation are

$$\omega_{\pm}\left(\Delta d_0\right) \simeq \frac{1}{2}\left(\omega_{m_1}^{(1)} + \omega_{m_2}^{(2)}\right) \pm \sqrt{\frac{\left(\omega_{m_1}^{(1)} - \omega_{m_2}^{(2)}\right)^2}{4} + K_h^2\left(\Delta d_0\right)}\,. \tag{S41}$$

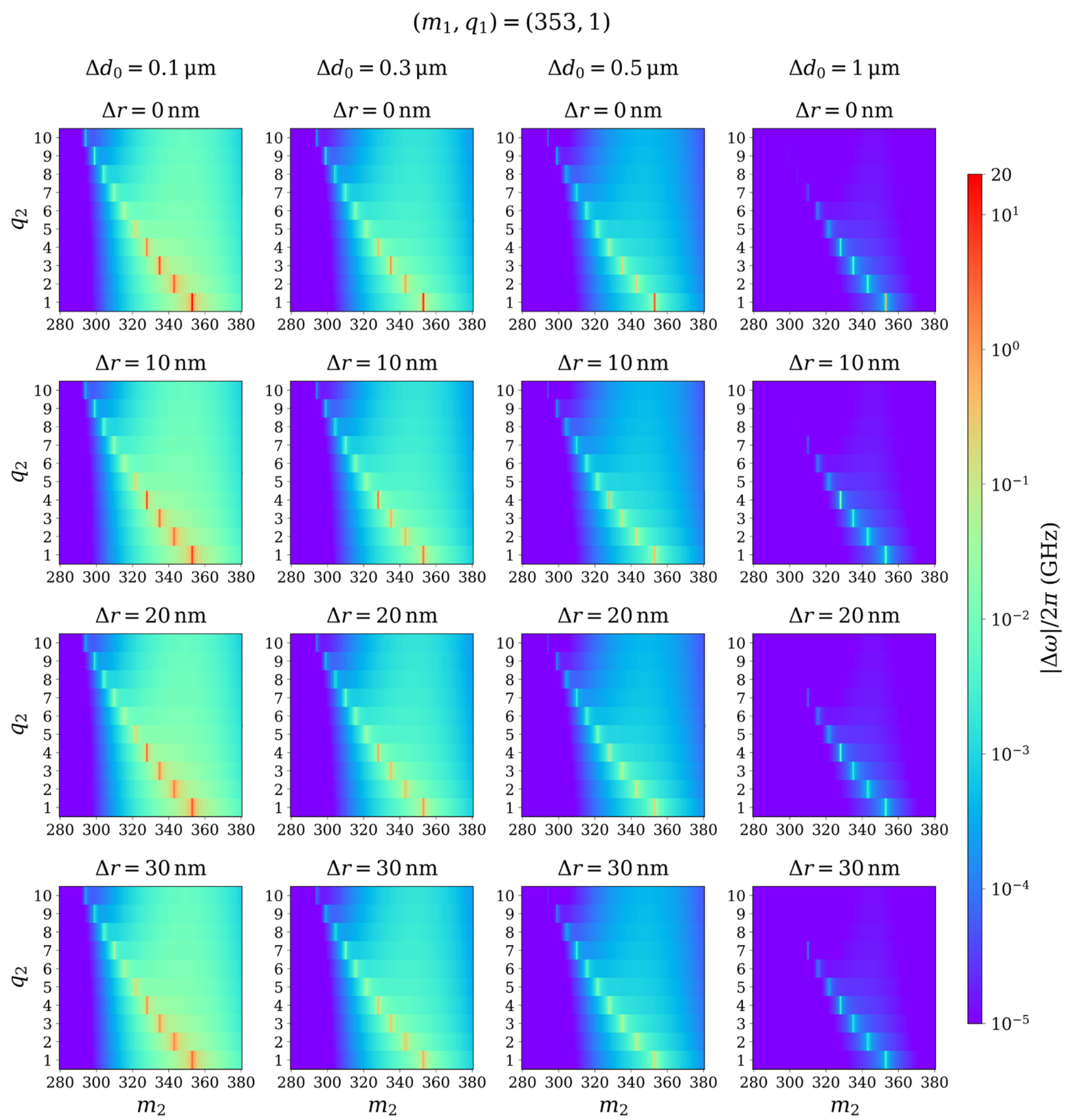

Fig. S5. the magnitudes $|\Delta\omega|/(2\pi)$ of the frequency shifts of the $(m_1, q_1) = (353,1)$ mode of fiber 1 induced by coupling to individual modes (m_2, q_2) of fiber 2.

Neglecting the much smaller second term in brackets in the expression for $T_{ij,m_i m_j}^{(h)}$ in Eq. (S37), which is proportional to h, and the small difference between the isolated and mean frequencies yields

$$K_h\left(\Delta d_0\right) \simeq K\left(\Delta d_0\right) = \frac{2i\omega_j}{\pi s_1 s_2 \sqrt{\left(n_1^2 - 1\right)\left(n_2^2 - 1\right)}} \frac{H_{m_1+m_2}^{(1)}\left(k_0\left(r_{01} + r_{02} + \Delta d_0\right)\right)}{H_{m_1}^{(1)}\left(s_1\right) H_{m_2}^{(1)}\left(s_2\right)}, \quad s_j = k_0 r_{0j}. \tag{S42}$$

This expression for the coupling, expressed through the cylindrical functions, is called here the *cylindrical approximation*.

In the large-order approximation we have $H_N^{(1)}(Nt) \simeq -i\left[2/\left(\pi N\sqrt{1-t^2}\right)\right]^{1/2} e^{N\eta(t)}$, where $\eta(t) = \operatorname{arcosh}(1/t) - \sqrt{1-t^2}$ [S7] and Eq. (S42) can be simplified further. In particular, in the first order in $\Delta d_0/d_0$, we find

$$\left|K\left(\Delta d_0\right)\right| \simeq \frac{\omega_j}{s_1 s_2\sqrt{\left(n_1^2-1\right)\left(n_2^2-1\right)}}\left[\frac{2m_1 m_2}{\pi\left(m_1+m_2\right)}\right]^{1/2}\left[\frac{\left(1-t_1^2\right)\left(1-t_2^2\right)}{1-t_0^2}\right]^{1/4}\exp\left(-\left(\left(\frac{m_1+m_2}{r_{01}+r_{02}}\right)^2-k_0^2\right)^{1/2}\Delta d_0\right). \quad \text{(S43)}$$

where

$$t_j = \frac{s_j}{m_j} < 1, \quad t_0 = \frac{k_0\left(r_{01}+r_{02}\right)}{m_1+m_2} < 1. \quad \text{(S44)}$$

For almost equal fibers, we set $m_j = m \simeq n_0 k_0 r_0$, and the exponent in this expression is reduced to $\exp\left(-k_0\sqrt{n_0^2-1}\Delta d_0\right)$. The latter approximation, which we call here the *planar approximation*,

$$\left|K\left(\Delta d_0\right)\right| \simeq \frac{c^{3/2}}{\pi^{1/2} r_0^{3/2}\omega_0^{1/2}\left(n_0^2-1\right)^{3/4}}\exp\left(-\frac{\omega_0}{c}\sqrt{n_0^2-1}\Delta d_0\right). \quad \text{(S45)}$$

coincides, after rescaling by the factor $2n_0^2/\omega_0$, with the dimensionless coupling I_{12} defined by Eq. (7) of the main text and considered in section S2. For comparison of analytical cylindrical (Eq. (S42)) and planar (Eq. (S45)) approximations with numerical calculations using the multipole method described in section S4, we set $r_{01} = 62.5\ \mu\text{m}$, $n_1 = n_2 = 1.44$, and $r_{02} = r_{01} + \Delta r$ with $\Delta r = 0,\ 10,\ 20,\ 30\ \text{nm}$. At the first radial quantum number, $q_1 = 1$, we follow the pair of eigenfrequencies associated with $m_1 = m_2 = 353$ as Δd_0 changes from 2 μm to 0.01 μm. In our numerical calculations we consider truncations with $L = 20,\ 30,\ 40,\ 50$ (see section S4). Their comparison confirms the calculated eigenfrequency accuracy relative to the change of their separation, which is better than 2% for $\Delta d_0 > 0.1$ μm, and better than 1% for $\Delta d_0 > 0.2$ μm.

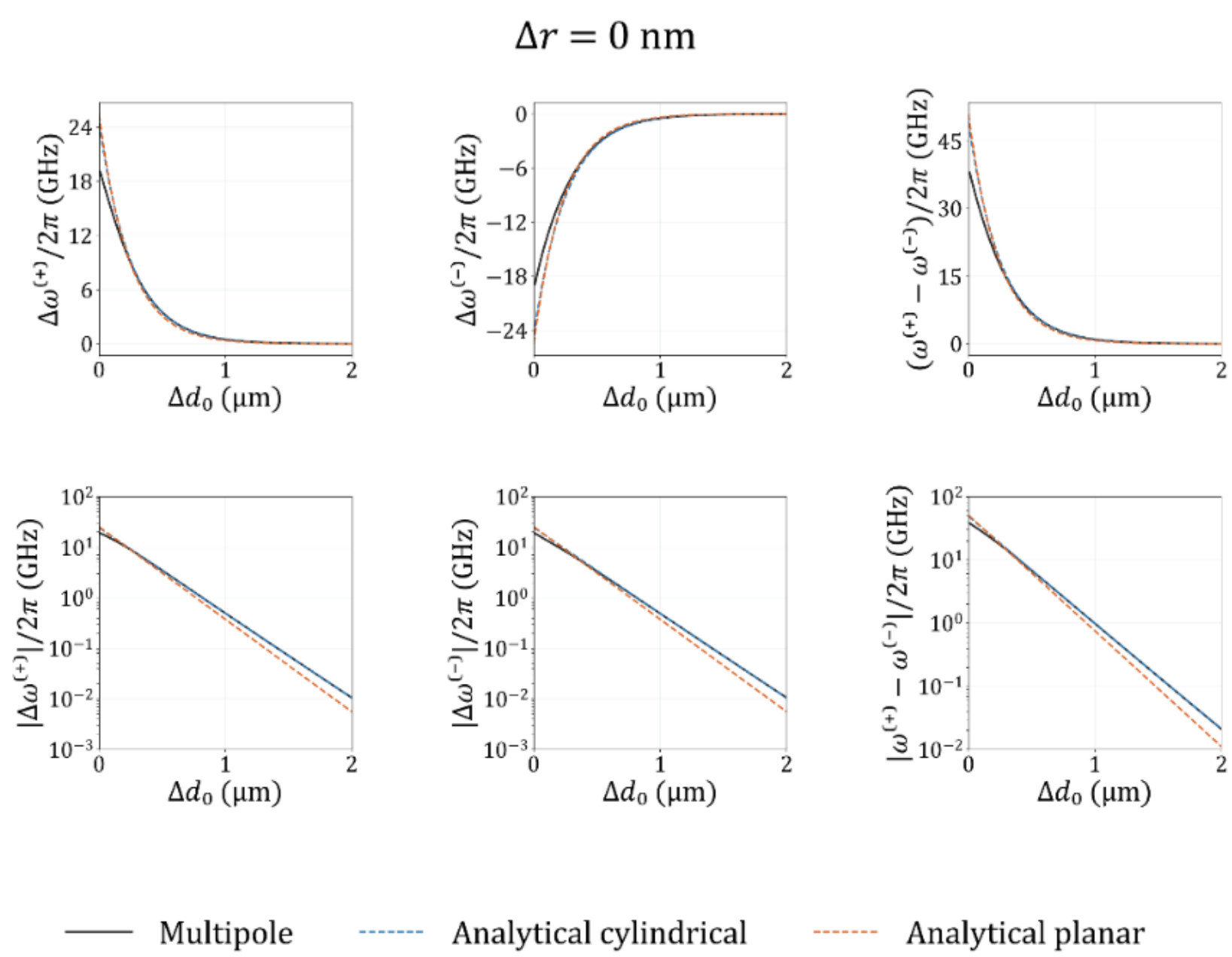

Fig. S6. Cutoff-frequency shifts $\Delta\omega^{(+)}/2\pi$ and $\Delta\omega^{(-)}/2\pi$ and the splitting $\left(\omega^{(+)}-\omega^{(-)}\right)/2\pi$ as functions of the fiber separation Δd_0 for identical fibers ($\Delta r = 0$): linear (upper row) and the corresponding logarithmic (lower row) scales.

Figure S6 shows the coupling-induced cutoff-frequency shifts $\Delta\omega^{(+)}/2\pi$ and $\Delta\omega^{(-)}/2\pi$ and the splitting $\left(\omega^{(+)}-\omega^{(-)}\right)/2\pi$ as functions of the fiber separation Δd_0 for identical fibers ($\Delta r = 0$) calculated by multipole method and compared with cylindrical and planar analytical approximations. The upper and lower rows show the results on linear and logarithmic scales, respectively. Absolute values are used in the logarithmic plots. Although the two analytical

predictions are close at small gaps, both overestimate the numerical splitting. At $\Delta d_0 = 0.01$ µm, the numerical, cylindrical, and planar splittings are 37.92, 47.34, and 48.87 GHz, respectively, giving analytical overestimates of approximately 25% and 29%. The cylindrical approximation improves rapidly with increasing separation: over gaps of 1-2 µm, its splitting and individual shifts differ from the numerical results by less than 0.03% and 0.6%, respectively. The planar expression decays more rapidly and underestimates the splitting by approximately 24-47% over the same interval.

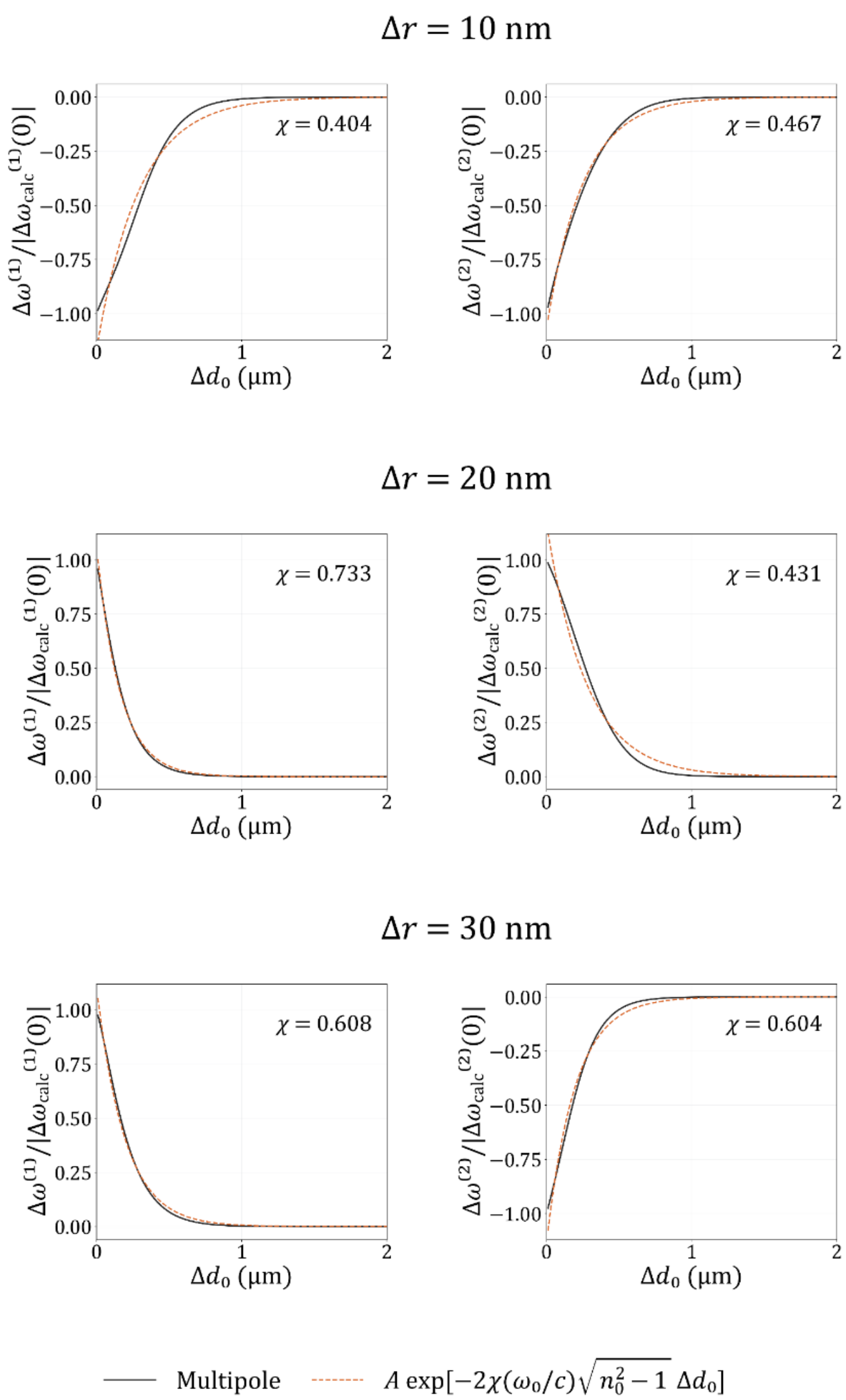

Fig. S7. Comparison of normalized cutoff-frequency shifts calculated by multipole method with fitted exponential dependencies for unequal fibers with radius differences $\Delta r = 10,\ 20,\ 30$ nm.

Figure S7 compares multipole calculations with fitted exponentials for the individual cutoff-frequency shifts of unequal fibers with radius differences $\Delta r = 10,\ 20,\ 30$ nm. The cylindrical and planar analytical approximations do not reproduce these shifts throughout the gap range considered. We therefore fit each branch independently to $A\exp\left(-2\chi(\omega_0/c)\sqrt{{n_0}^2 - 1}\Delta d_0\right)$. Here $\chi = 0.5$ corresponds to the eigenfrequency splitting in the planar approximation (Eq (S45)) when the eigenfrequencies of uncoupled modes are equal, while $\chi = 1$ corresponds to the case when the isolated eigenfrequencies are different and their splitting is much smaller than their separation in isolated fibers. Each curve is normalized by the magnitude of its numerical shift at the smallest calculated gap. The fits cover gaps of 0.01-2 µm and yield $\chi = 0.404 - 0.733$, quantifying differences in the effective gap dependence among the branches and radius mismatches. Across the six curves, the root-mean-square fit errors are 0.8-3.8% of the maximum shift magnitude, with maximum deviations of up to 15%. This comparison will be used for estimating the experimental parameters of microresonators induced by tilted fibers discussed in the main text.

S6. Squeeze-induced cutoff frequency shift

Here we derive analytical estimates for two identical silica fibers squeezed symmetrically at their contact. We consider the 2D model, when the values of cutoff frequencies coincide with values of 2D eigenfrequencies.

We start with the purely *geometrical effects of local fiber deformation*. Let $\xi > 0$ be the total reduction of the center-to-center distance due to squeezing. In the flat-contact approximation, we assume that a circular cap of height $\xi/2$ is removed from each fiber and replaced by a straight contact segment [S8] (Fig. S8). For $\xi/r_0 \ll 1$, the contact half-width $a = \sqrt{r_0\, \xi}$. Using the WGM quantization condition $\omega n_0 L_f/c = 2\pi m$, where $m \gg 1$ is an integer and $L_f = 2\pi r_0$ is the fiber circumference, we determine the frequency shift due to the change of the circumference ΔL_f as $\delta\omega_{\text{geom}} = -\omega_0 \Delta L_f/L_f$.

For one fiber, the circular arc $2r_0\theta$ is replaced by the straight segment $2a = 2r_0\sin(\theta)$. For both fibers, the total circumference and its change are therefore $2L_f = 4\pi r_0$ and $2\Delta L_f = 2(2a - 2r_0\theta) = 4r_0(\sin(\theta) - \theta)$. Expanding $\sin(\theta)$, we find the frequency shift caused by geometric fiber deformation

$$\delta\omega_{\text{geom}} \simeq \frac{\omega_0}{6\pi}\left(\frac{\xi}{r_0}\right)^{3/2} \tag{S46}$$

The frequency shift determined by this equation is positive because replacing an arc by a chord (Fig. S8) shortens the optical path.

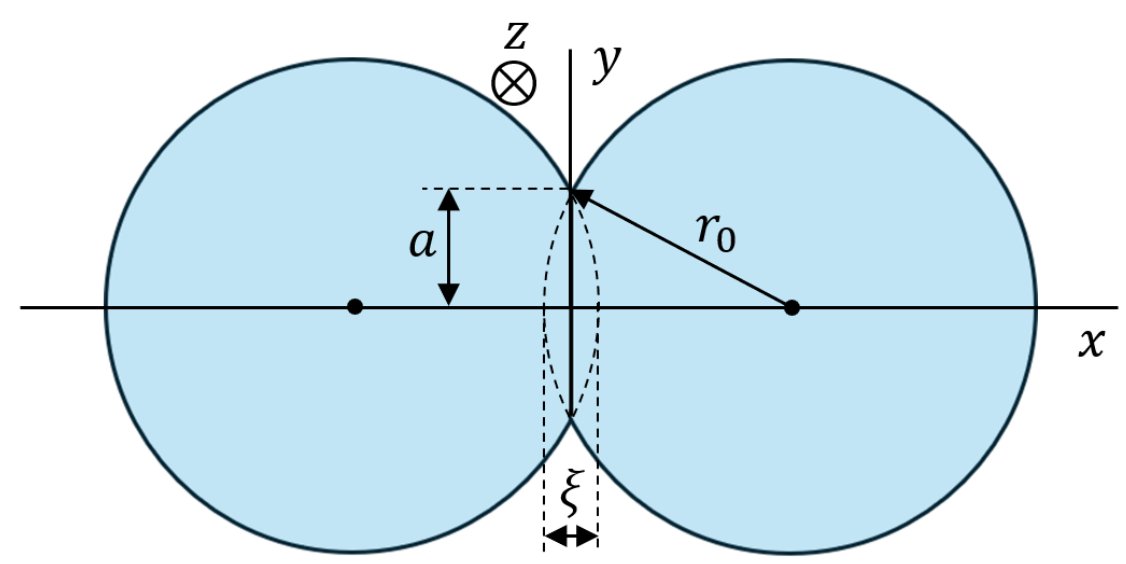

Fig. S8. Cross-section of identical fibers with radius r_0 squeezed by ξ.

Let us now consider the effects of the *photoelastic change in refractive index*. We choose axis x normal to the contact surface, y parallel to the contact surface, and z along the fiber axis and along the electric field of the two-dimensional TE mode (Fig. S8). For isotropic silica, the index change for this polarization is $\Delta n_z = -\frac{1}{2}n_0^3\left[p_{11}S_{zz} + p_{12}\left(S_{xx} + S_{yy}\right)\right]$ where S_{ij} are strain components. Here p_{11} and p_{12} are the strain-optic coefficients. In the simplest uniaxial estimate, $S_{xx} = S$ and the free lateral strains are $S_{yy} = S_{zz} = -\mu S$, where μ is the Poisson ratio. The average compressive strain is estimated as the indentation of one fiber divided by the contact depth a: $S \simeq -\xi/2a \simeq -\frac{1}{2}\sqrt{\xi/r_0}$. Consequently, we have

$$\Delta n_z \simeq \frac{n_0^3}{4}\sqrt{\frac{\xi}{r_0}}P_z, \quad P_z = p_{12}\left(1-\mu\right) - p_{11}\mu. \tag{S47}$$

For silica fibers, we have $p_{11} = 0.121$, $p_{12} = 0.270$, and $\mu = 0.17$ give $P_z = 0.2035$ [S9].

Let $E_j^{(0)}(\mathbf{r})$ be the electric field of the unperturbed exchange supermode $j = +$ or $j = -$, and let Ω_s denote the strained region inside the two fibers. For a refractive index perturbation $\Delta n_z(\mathbf{r})$ in silica fibers, first-order eigenfrequency perturbation theory gives for the photoelastic eigenfrequency shift

$$\delta\omega_j^{\text{PE}} = -\frac{\omega_0 n_0}{U_j}\int_{\Omega_s}\Delta n_z\left(\mathbf{r}\right)\left|E_j^{(0)}\left(\mathbf{r}\right)\right|^2 dA, \quad U_j = \int n^2\left(\mathbf{r}\right)\left|E_j^{(0)}\right|^2 dA. \tag{S48}$$

Here U_j is the electric field normalization and $n(\mathbf{r}) = n_0$ inside fibers and $n(\mathbf{r}) = 1$ outside them. If the index change is replaced by its mean value $\overline{\Delta n_z}$ in Ω_s, define the unperturbed modal-energy fraction

$$\delta\omega_j^{\mathrm{PE}} \simeq -\omega_0 \frac{\overline{\Delta n_z}}{n_0} \eta_j, \quad \eta_j = \frac{n_0^2}{U_j} \int_{\Omega_s} \left| E_j^{(0)} \right|^2 dA. \tag{S49}$$

For symmetric weak coupling, the leading self-energy fractions of the two exchange parities are equal: $\eta_+ \simeq \eta_-$.

The fundamental radial WGM can be expressed through the Airy function as described in Section S1 which yields $\eta_j \simeq 2n_0^2\xi/[\pi r_0(n_0^2-1)]$ and

$$\delta\omega_{\mathrm{PE}} \simeq -\frac{n_0^4 \omega_0 \left[p_{12}(1-\mu) - p_{11}\mu \right]}{2\pi\left(n_0^2-1\right)} \left(\frac{\xi}{r_0} \right)^{3/2}. \tag{S50}$$

Adding the geometric and photoelastic contributions defined by Eqs. (S46) and (S50), we find the total frequency shift

$$\delta\omega \simeq \frac{\omega_0}{\pi} \left(\frac{\xi}{r_0} \right)^{3/2} \left\{ \frac{1}{6} - \frac{n_0^4 \left[p_{12}(1-\mu) - p_{11}\mu \right]}{2\left(n_0^2-1\right)} \right\}. \tag{S51}$$

Substituting the parameters for the silica fibers given above, we find within an order of magnitude

$$\delta\omega \simeq -0.1\omega_0 \left(\frac{\xi}{r_0} \right)^{3/2}. \tag{S52}$$

Remarkably, the change of the exchange splitting is predominantly determined by the geometrical changes for finite ξ. To determine this change, we introduce the gap between unsqueezed fibers along coordinate x near the contact point before squeezing, $\Delta h_0(y) = y^2/r_0$, and after squeezing (Fig. S8):

$$\Delta h_\xi(y) = \max\left(\frac{y^2}{r_0} - \xi, 0 \right) \tag{S53}$$

Replacing the coordinate z by the variable ξ in Eq. (S21) for the overlap integral, $I_{12}(z) \rightarrow I_{12}(\xi)$, and introducing the frequency splitting $\Delta\omega(\xi)$, we find for $\kappa\xi \ll 1$

$$\frac{I_{12}(\xi)}{I_{12}(0)} = \frac{\Delta\omega(\xi)}{\Delta\omega(0)} = \left(\int_{-\infty}^{\infty} e^{-\kappa \Delta h_\xi(y)} dy \right) / \left(\int_{-\infty}^{\infty} e^{-\kappa \Delta h_0(y)} dy \right) \simeq 1 + \kappa\xi, \quad \kappa = \frac{\omega_0}{c}\left(n_0^2-1\right)^{1/2}. \tag{S54}$$

Using the expression for the asymptotic splitting found from Eqs. (S41) and (S45)

$$\Delta\omega(0) \simeq \frac{2c^{3/2}}{\pi^{1/2} r_0^{3/2} \omega_0^{1/2} \left(n_0^2-1\right)^{3/4}}, \tag{S55}$$

we find

$$\delta\omega = \kappa\xi\Delta\omega(0) = \frac{2(c\omega_0)^{1/2}}{\pi^{1/2} r_0^{3/2} \left(n_0^2-1\right)^{1/4}} \xi. \tag{S56}$$

Using $r_0 = 62.5$ μm, $n_0 = 1.44$, and $\omega_0 = 2\pi \times 193$ THz, we obtain an evanescent decay rate $\kappa \cong 4.2$ $\mu\mathrm{m}^{-1}$. Thus, Eq. (S54) predicts an increase in the inter-fiber coupling of approximately 0.4–4% for symmetric squeezing by ξ = 1–10 nm. With an unsqueezed full CF splitting $\Delta\omega(0)/(2\pi) \simeq 50$ GHz, the corresponding increase in splitting is approximately 0.2–2 GHz. Each CF shifts by half of this change, with opposite signs for the two branches. The geometric and photoelastic contributions from Eqs. (S46) and (S50) partly cancel, giving a much smaller common midpoint shift of approximately −1 MHz for 1 nm squeezing and −30 MHz for 10 nm squeezing. These estimates therefore indicate that squeezing by 1–10 nm can induce CF shifts of approximately 0.1–1 GHz, comparable to the observed discrepancy. The numerical values are approximate and depend on the simplified local contact model.

References

S1. A. V. Snyder and J. D. Love, *Optical waveguide theory*. (London, Chapman and Hall, 1983).

S2. M. Sumetsky, Optical microfiber coil delay line. *Opt. Express* **17**, 7196-7205 (2009).

S3. M. Sumetsky, Theory of SNAP devices: basic equations and comparison with the experiment. *Opt. Express* **20**, 22537–22554 (2012).

S4. L. D. Landau and E. M. Lifshitz, *Quantum Mechanics, Nonrelativistic Theory. Course of theoretical physics, vol. III* (Pergamon Press, 1977).

S5. G. O. Olaofe, Scattering by an arbitrary configuration of parallel circular cylinders. *J. Opt. Soc. Am.* **60**, 1233–1236 (1970)

S6. E. N. Bulgakov, K. N. Pichugin, and A. F. Sadreev, "Evolution of the resonances of two parallel dielectric cylinders with distance between them," *Physical Review* A 100, 043806 (2019).

S7. NIST *Digital Library of Mathematical Functions*, https://dlmf.nist.gov.

S8. K. L. Johnson, *Contact Mechanics* (Cambridge University Press, 1985).

S9. M. J. Weber, *Handbook of Optical Materials* (CRC Press, Boca Raton, FL, 2003).
